# Specific and Non-specific Factors of Animal-Assisted Interventions Considered in Research: A Systematic Review

**DOI:** 10.3389/fpsyg.2022.931347

**Published:** 2022-06-28

**Authors:** Cora Wagner, Carmina Grob, Karin Hediger

**Affiliations:** ^1^Division of Clinical Psychology and Psychotherapy, Faculty of Psychology, University of Basel, Basel, Switzerland; ^2^Division of Clinical Psychology and Animal-Assisted Interventions, Faculty of Psychology, University of Basel, Basel, Switzerland; ^3^REHAB Basel, Clinic for Neurorehabilitation and Paraplegiology, Basel, Switzerland; ^4^Department of Epidemiology and Public Health, Human and Animal Health Unit, Swiss Tropical and Public Health Institute, Allschwil, Switzerland; ^5^Faculty of Psychology, Open University, Heerlen, Netherlands

**Keywords:** specific factor, contextual factor, mechanism, systematic review, animal-assisted intervention

## Abstract

Research on animal-assisted interventions (AAIs) has increased massively in the last few years. But it is still not clear how AAIs work and how important the animal is in such interventions. The aim of this systematic review was to compile the existing state of knowledge about the working mechanisms of AAIs. We searched 12 major electronic databases for previous AAI studies with active control groups. Of 2001 records identified, we included 172 studies in the systematic review. We extracted previously published hypotheses about working mechanisms and factors that have been implicitly considered specific or non-specific in AAI research by categorizing control conditions using content analysis. We analyzed the categories using descriptive statistics. We found that 84% of the included studies mentioned a hypothesis of working mechanisms, but 16% did not define specific hypotheses. By analyzing their control conditions, we found that in most controlled studies, the animal or the interaction with the animal was implicitly considered as a specific factor for the effects of the AAI. Non-specific factors such as therapeutic aspects, social interactions, or novelty have also been controlled for. We conclude that AAI research still cannot answer the question of how and why AAIs work. To address this important research gap, we suggest using component studies with innovative control conditions and results from placebo research to address both the specific and non-specific, contextual factors of AAIs to disentangle its mechanisms.

**Systematic Review Registration:**

https://www.crd.york.ac.uk/prospero/display_record.php?RecordID=158103, identifier: CRD42020158103.

## Introduction

Research on animal-assisted interventions (AAIs) has increased massively in the last few years (Rodriguez et al., 2021). But it is still not clear how important the animal is in such interventions. In 2012, Marino addressed construct validity in AAIs and concluded in a review that it is a hugely neglected topic (Marino, 2012). One decade later, the evidence of the effectiveness of AAIs is increasing (Waite et al., 2018; Wood and Fields, 2019; Borgi et al., 2020; Babka et al., 2021; Chang et al., 2021; Diniz Pinto et al., 2021; Hediger et al., 2021; Nieforth et al., 2021), but the question of construct validity is still unresolved. Previous research has mainly focused on investigating *if* AAIs work but almost entirely ignored the question of *how* it works. The claim that the underlying mechanisms of AAIs are not clear is not new, but it is intensifying, and researchers are debating the internal validity of a broad range of different interventions that are all subsumed under the umbrella term of AAI (Kazdin, 2017; Serpell et al., 2017; López-Cepero, 2020; Rodriguez et al., 2021).

AAIs are based on the assumption that the animal is the key relevant component for the effects of such interventions. It has been proposed that an animal adds something different to a therapeutic setting compared to a human or another stimulus. The literature has therefore claimed that a live animal is a highly specific component of AAIs (Marino, 2012). It is, however, still unclear if the living animal itself—and if so, what specific characteristics of the animal—leads to the documented effects of AAIs. Specificity is a major challenge in current AAI research, so it is crucial to identify if the effects of AAIs are due to the presence of an animal specifically.

López-Cepero (2020) proposed a component-centered approach to investigate how AAIs work. AAIs consist of a complex mixture of components such as being confronted with a novel stimulus and situation, receiving increased attention from a therapist, engaging in increased physical activity and physical contact, or sometimes even being in a different environment. AAI should thus be seen as a treatment (such as psychotherapy, speech therapy, or physiotherapy) or even as a specific manualized therapy (such as cognitive behavioral therapy, for example) with the addition of a specific component: the animal. We agree with this approach of disentangling the effect of different treatment components, but we propose going even a step further by using a component-centered approach to look at the animal, the added component. The animal itself is a complex stimulus with different characteristics (Marino, 2012; Rodriguez et al., 2021): for example, animals react to clients' behavior, move proactively, have fur or feathers, come in different shapes and colors, and have varying temperaments and personalities. All of these characteristics could lead to different effects.

Component studies are the best method for examining the active components of a treatment (Cuijpers et al., 2019). Their study designs can decompose multicomponent treatments by comparing the complete intervention with an intervention in which one component is left out (dismantling studies) or with an intervention with an additional component (additive studies) (Bell et al., 2013; Mira et al., 2019). The effects of an intervention can be distinguished into specific effects and contextual, or non-specific, effects (Wampold, 2021). Specific effects are effects that are caused by the specific intervention, while contextual, or non-specific, effects result from factors that are not specific to the intended intervention and that appear in every intervention, such as treatment expectations, the therapeutic alliance (Rossettini et al., 2018; Wampold, 2021), novelty, demand characteristics, and effects from experimenters' expectations (Marino, 2012). Such non-specific effects are considered as confounding variables that can affect internal and external validity (Carlino et al., 2011; Geers and Miller, 2014).

It is crucial that we begin to understand what makes AAIs effective. To pursue this goal, we must know what mechanisms, specific factors, and non-specific factors have been investigated so far. While older studies usually did not control for non-specific effects, recent studies have started to dismantle the potential components of AAIs and even of the animal by using more specific and rigorous controls. Investigating the used control conditions in previous AAI studies makes it possible to infer the authors' assumptions about the specific and non-specific effects of AAIs.

The aim of this systematic review was to compile the existing state of knowledge about how AAIs work. To do so, we collected the explicitly stated hypotheses about the working mechanisms of AAIs mentioned in previous studies and compared the control condition with the experimental condition of previous AAI studies in order to derive which implicit specific and non-specific factors of AAI have been considered to be relevant so far.

## Methods

### Search Strategy

We conducted a systematic literature search in the following databases: PsychINFO, PSYNDEX, ERIC, MEDLINE, Embase, PubMed, Cochrane Library, Web of Science, Scopus, CINAHL, PTSDpubs, and Dissertations and Theses. A summary of the applied search strategies can be found in Appendix Table S1. We also used other sources to identify studies.

We imported all the records into Covidence, a systematic review software (Veritas Health Innovation, Melbourne, Australia), where duplicates were identified and removed. The screening was also performed in Covidence. The titles and abstracts of the included records were screened by two independent researchers in duplicate to exclude obvious irrelevant references and duplicates. Full texts were again screened by two independent researchers in duplicate to examine the records in more detail for inclusion and exclusion criteria. Conflicts were resolved by consensus among all the researchers involved in the screening process (CW, KH, and CG).

Identifying, screening, and determining the eligibility of the studies was done according to the Preferred Reporting Items for Systematic Reviews and Meta-Analyses (PRISMA) (McInnes et al., 2018). The study procedure was defined a priori, and the protocol was preregistered with PROSPERO (registration number: CRD42020158103). The date of the last search was January 13, 2022.

### Study Selection

We used the PICO elements *Intervention* and *Comparison* to include relevant studies (EUnetHTA, 2019; Frandsen et al., 2020). The elements *Population* and *Outcomes* were irrelevant for this review (all were included). To be eligible for inclusion, studies had to (1) investigate an AAI (*Intervention*), (2) include an active control group (*Comparison*), and (3) be written in English or German.

**Figure 1:**
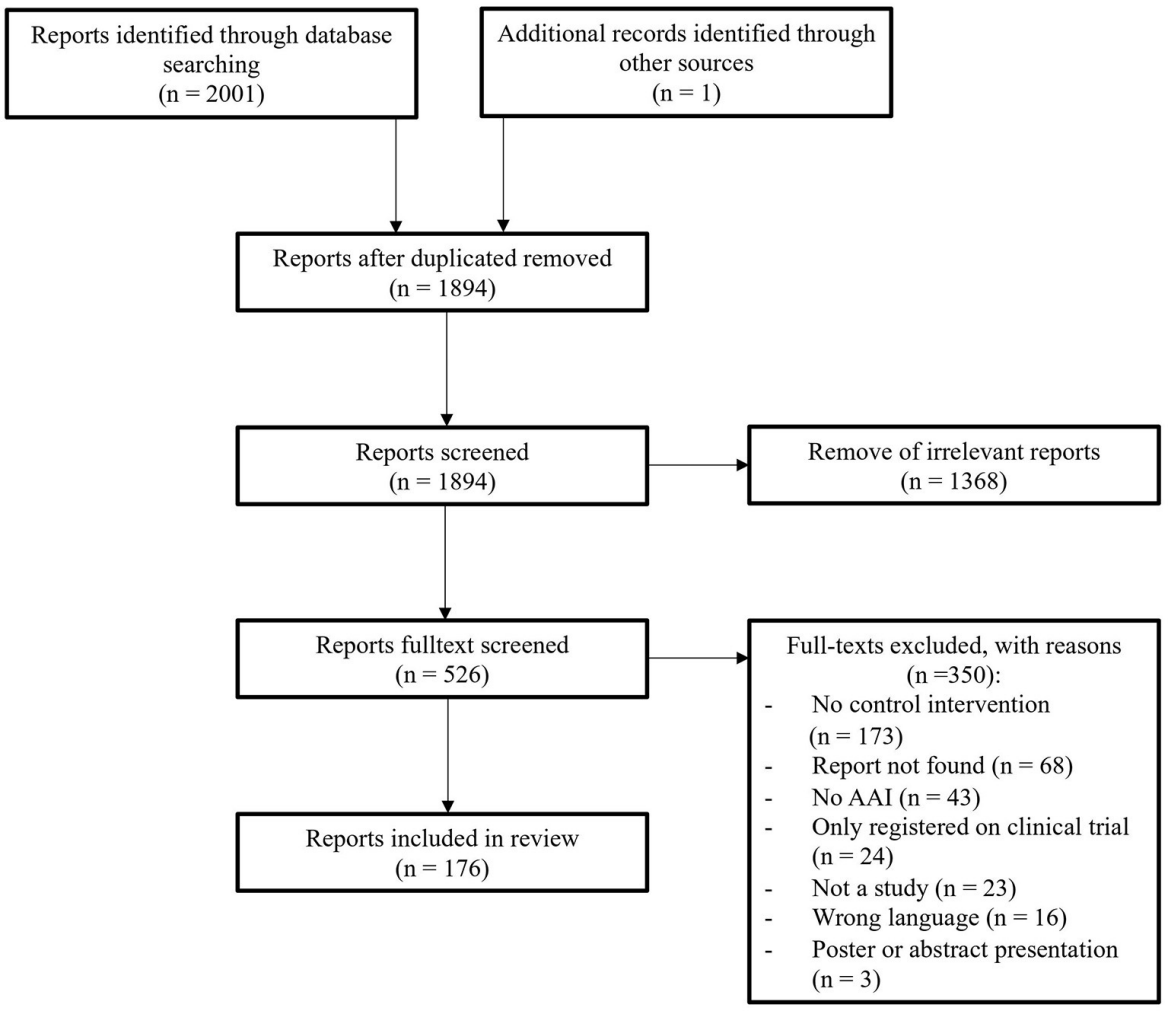
Flow chart.

We included all studies that examined a type of AAI (e.g., animal-assisted therapy, animal-assisted activity, animal-assisted education, hippotherapy, pet therapy) with a live animal (*Intervention*). We followed the terminologies of the IAHAIO (2018) and included every study with an intervention that can be considered an AAI according to the IAHAIO definition. We excluded studies on pet ownership. We included all type of study design as long there was an active control group (i.e., randomized controlled trial, cross-over study) (*Comparison*). We included all forms of active control conditions. Active control was defined as a condition in which the participants received a specific intervention offered by the study team. We excluded studies where participants in the control condition received standard care (i.e., care that was not offered by the study team), where they were on a waiting list, or where the study was a pre–post design with only one group. Further, we excluded records that were only registered as clinical trials and abstracts or poster presentations, because they did not provide sufficient information for our review. We contacted the study authors if a record was not available through university libraries. Studies were excluded if we were not able to receive the full text (see Figure 1 for the flow chart).

We first screened the titles and abstracts of the records. During full-text screening we excluded all records that did not fulfill all our inclusion criteria.

### Data Extraction

Prior to the data extraction, all researchers received training in using the form for extracting information on the following categories: first author's name and country, publication year, the characteristics of the experimental and control intervention, factor hypotheses, and the animal included in the study.

In a first step, all the data were independently extracted and coded in duplicate by a team of five research assistants in Microsoft Office Excel 2016. In a second step, all disagreements between the two raters were identified independently by two researchers, and conflicts were resolved by consensus among all the researchers involved in the screening process (CW, KH, and CG).

### Data Analyses

To extract the factor hypotheses, the specific factors, and the non-specific factors, we used structured qualitative content analysis following Mayring (2014). Two independent raters analyzed the manuscripts independently in a first step and extracted the hypotheses, the specific factors, and the non-specific factors. The content was reduced to units of meaning that were then consolidated to items. In a second step, the two coding schemes were compared, disagreements were discussed with two authors (CW and KH), and consensus was reached on one scheme. One author (CG) defined superordinate categories for the items of the extracted hypotheses, the specific and non-specific factors. These proposed superordinate categories were then discussed with the other two authors (CW and KH) and adjusted. All hypotheses and factors that were not mentioned more than twice and did not fit into any existing category were classified as “other.”

We analyzed the categories using descriptive statistics. The base rate for the study characteristics, factor hypotheses, and specific and non-specific factors was the total number of the included studies (*N* = 172). Descriptive analyses were carried out using R for Mac, version 1.4.1103.

#### Factor Hypotheses

We defined factor hypotheses as hypotheses, factors, or mechanisms that authors mentioned in the introductions of their studies to explain how AAIs work. It was possible for a study to mention several hypotheses. Two independent raters independently extracted factor hypotheses in the studies. All disagreements were solved by two authors (CW and KH). After that, two authors (CW and KH) reviewed the categories of the factor hypotheses and subsumed them into 11 main categories.

#### Specific Factors of AAIs

We defined a factor as specific if it was present in the experimental condition but not in the control condition. Two raters independently compared the characteristics of the experimental interventions and the control interventions. All factors that were not present in the control conditions were coded as specific factors. The two raters extracted the factors independently. After that, they independently summarized the factors into categories. All disagreements were resolved by a third rater (CW). Then two researchers (CW and KH) reviewed the categories and subsumed then into nine main categories.

Items were listed in several categories if they were applicable. For example, the item *training in animal care* was included in category 5, “taking care of an animal,” because aspects of taking care of an animal were present and in category 8, “education about an animal,” because subjects received training (see **Table 2**; **Figures 3**, **4**).

#### Non-specific Factors of AAIs

All factors that existed in both the experimental and the control interventions were defined as non-specific factors. Two independent raters compared the experimental and control conditions from each study and independently listed all the factors that occurred in both interventions. In a second step, they independently categorized the factors. All disagreements were then resolved by a third rater (CW). After the disagreements were resolved, two authors (CW and KH) reviewed the categories of non-specific factors and subsumed them into 14 main categories.

It was possible for an item to be listed in several categories. *Physiotherapy*, for example, was included in category 1, “physical activity,” but also in category 2, “therapeutic aspects.” Moreover, it was possible to code the same item as both specific and non-specific factor. The reason for this is because it was possible that in one study a factor was considered as specific and in another study as a non-specific factor depending on the study design. For example, if the animal was only present in the experimental condition but not in the control condition in one study, we categorized “animal” as a specific factor. However, if the animal was also present in the control condition, then “animal” was categorized as a non-specific factor.

## Results

### Search Results

We identified 2,001 reports and screened 1,893 titles and abstracts after we had removed duplicates. We assessed the full text of 525 reports for eligibility. In the end, 172 studies, which were published in 176 reports, fulfilled our inclusion criteria and were included in this systematic review (see Figure 1).

### Study Characteristics

The included studies were published in records between 1987 and 2022. Of these, 76.14% (*n* = 134) were published between 2014 and 2022; 164 were peer-reviewed and published as journal articles, and only six were not published.

The majority of the reports (*n* = 116) were conducted in the USA (*n* = 74), Germany (*n* = 13), South Korea (*n* = 12), Spain (*n* = 9), or Italy (*n* = 8). Regarding the animals, a large majority of the studies used dogs (*n* =107) or horses (*n* = 50), followed by cats (*n* = 7), guinea pigs (*n* = 6), or farm animals (*n* = 6) such as donkeys, goats, sheep, chickens, pigs, and rabbits (see Table 1 for an overview of the study characteristics).

**Table 1:** Study characteristics.

**First author**	**Year**	**Country**	**Type of publication**	**Animals**	**Control condition**	**Intervention**
Abdel-Aziem	2022	Saudi Arabia	Journal article	Horse	Physiotherapy (Schroth exercises)	Hippotherapy plus home workout exercises
Alemdaroglu	2016	Turkey	Journal article	Horse	Conventional rehabilitation	Horseback riding plus therapist-directed exercises
Allen	2021	USA	Journal article	Dog	Trauma-Focused Cognitive-Behavioral Therapy (TF-CBT)	TF-CBT with AAT as adjunctive therapy
Ambrozy	2017	Poland	Journal article	Horse	Physical education classes	Horse's walk and horse's trot
An	2021	South Korea	Journal article	Dog	Gait traininig	Gait training with dog
Antonioli	2005	Honduras	Journal article	Dolphin	Outdoor nature program (water activities)	Play, swim, and take care of the animals
Aranda-Garcia	2015	Spain	Journal article	Horse	Two control condition: (a) traditional exercise program or (b) CG: none	Fun-oriented exercise and body workouts involving the horse
Ashtari	2018	Iran	Journal article	Dolphins	Training and playing in water	Interaction and swimming with dolphins
Asqarova	2021	USA	Dissertation	Guinea pig	Reading session	Guinea pig therapy
Bachi	2014	USA	Dissertation	Horse	Correctional and vocational programs	Equine-assisted intervetion
Bailey	1987	USA	Dissertation	Dog	Two control conditions: (a) structured curriculum about pets and pet care; (b) small group activities unrelated to the pet curriculum	A humane education curriculum guide and interaction with puppy
Banks	2008	USA	Journal article	Dog	Two control conditions: (a) visit of the robot dog AIBO or (b) no intervention	Sitting in chair or upright in bed with the dog next to the resident
Barak	2001	Israel	Journal article	Dog and cat	Reading and discussing news in group	Taking care of dog or cat
Barker	2020	USA	Journal article	Dog	Waiting room without animal	AAI with dog
Barker	2016	USA	Journal article	Dog	Attention-control condition (completing the Family Life-Space Diagram)	Free interaction with dog
Barker	2003	USA	Journal article	Dog	Reading magazines for 15 min	Conversation with dog handler, interaction with dog
Beck	2012	USA	Journal article	Dog	Occupational therapy life skills classes	Interaction with dog and obedience
Becker	2017	USA	Journal article	Dog	Social skills training	Animal-assisted Social Skills Training group activity with dog
Beetz	2012	Germany	Journal article	Dog	Two control conditions: trier social stress test with (a) toy-dog or (b) friendly female student	Trier social stress test in the presence of a dog
Beetz	2015	Germany	Journal article	Horse	Conventional play-based early intervention (PBI)	Riding and different activities on the horse
Beinotti	2013	Brazil	Journal article	Horse	Physiotherapy	Touching animal or reaching for an object
Beinotti	2010	Brazil	Journal article	Horse	Physiotherapy	Hippotherapy
Benda	2003	USA	Journal article	Horse	Sitting astride the barrel and watched a horse video	Horseback riding
Berget	2008	Norway	Journal article	Farm animals	Ordinary psychiatric treatment	Working with farm animals
Berry	2012	Italy	Journal article	Dog	Physical therapy/socialization group	Physical therapy session or social session with a dog
Bialoszewski	2011	Poland	Journal article	Horse	Home-based rehabilitation	Exercises with the horse at walk, trot, or while standing in place
Binfet	2022	USA	Journal article	Dog	Handler-only interaction	Canine assisted intervention with or without physical contact
Bowin	2020	USA	Dissertation	Dog	Cold pressor test without dog present	Cold pressor test with physical contact to dog afterwards
Boyer	2014	USA	Journal article	Cat	Toy cat activity	Interaction and taking care of cat
Branson	2017	USA	Journal article	Dog	Plush stuffed dog	Interaction with therapy dog
Bravo Gonçalves	2020	Brazil	Journal article	Horse	Walking alongside a horse	Hippotherapy with blanket or saddle mount
Breitenbach	2009	Germany	Journal article	Dolphins/ (farm animals)	Three control conditions: (a) interaction with dolphins, (b) farm animals or (c) no treatment	Dolphin assisted-therapy sessions (different stages: introduction, interaction, play, direct contact, swim)
Bunketorp	2012	Sweden	Journal article	Horse	Rhythm and music-based therapy	Therapeutic riding
Bunketorp	2019	Sweden	Journal article	Horse	Music-based therapy	Hippotherapy
Calvo	2016	Spain	Journal article	Dog	Choosing a single activity (art therapy, group sports, dynamic psycho-stimulation or gymnastics)	Interaction with therapy dog
Capparelli	2020	USA	Journal article	Dog	Interview	Interview with a dog in the room
Charnetski	2004	USA	Journal article	Dog	Two control conditions: (a) petting stuffed animal or (b) sitting comfortable on couch	Petting a real-life dog
Chen	2021	Taiwan	Journal article	Dog	Non-animal related intervention	AAT group with dog
Cho	2017	South Korea	Journal article	Horses	Mechanical horseback riding	Horseback riding
Clark	2020	USA	Journal article	Dog	Visit handler only	Visit of dog and handler
Cole	2007	USA	Journal article	Dog	Two control condition: (a) visit volunteer or (b) usual care	Patients may pet the dog and talk to the dog and volunteer
Colombo	2006	Italy	Journal article	Canary	Two control condition: (a) receiving plant or (b) receiving nothing	Look after canary
Costa	2019	Brazil	Journal article	Dog	Speech Therapy	Speech Therapy Program with Dog
Crossman	2015	USA	Journal article	Dog	Two control conditions: (a) viewing images of dog or (b) no treatment control	Free interaction with dog (petting, playing etc.)
Crump	2015	USA	Journal article	Dog	Study 1: non-stressful activities // Study 2: drawing activities.	Animal-assisted activity with a dog
Dietz	2012	USA	Journal article	Dog	Two control conditions: (a) no dog, (b) no story, dog present	Group therapies with dogs integrated in stories
Dunalp	2020	USA	Dissertation	Fish	Empathy-based mini lessons in classroom	Empathy-based lessons with pet fish
Eckes	2020	Germany	Journal article	Mice	Biology lessons	Care treatment and lesson with mice
El-Maniawy	2012	Egypt	Journal article	Horse	Designed excercise programm	Horseback riding
Fiocco	2017	Canada	Journal article	Dog	Relax in a seated position for 10 min	Free interaction with therapy dog
Flynn	2019	USA	Journal article	Dog	Intensive family preservation services	AAT as adjunctive to IFPS
Foerder	2021	USA	Journal article	Dog	Waiting with stuffed dog/waiting with research assistant	Waiting room with dog
Friedmann	2015	USA	Journal article	Dog	Attentional control intervention	Skills taught/reinforced with different components of the dog visit program include: feeding, brushing etc.
Funakoshi	2018	Japan	Journal article	Horse	Exercise using the horseback riding simulator	Horseback riding
Fung	2014	Hong Kong	Journal article	Dog	Identical play therapy procedure using a doll	Play therapy with a dog
Gabriels	2015	USA	Journal article	Horse	Barn activity	Therapeutic horseback riding
Gabriels	2018	USA	Journal article	Horse	Barn activity	Therapeutic horseback riding
Germone	2019	USA	Journal article	Dog	Novel toy and handler control	Animal-assisted activities in small groups
Gocheva	2018	Switzerland	Journal article	Suitable animal	Standard therapy session	AAT
Gee	2019	USA	Journal article	Fish	Two control conditions: (a) viewing plants and water; (b) viewing empty tank	Viewing fish tank
Grajforner	2017	UK	Journal article	Dog	Two control conditions: (a) interaction with the dog or (b) interaction with the handlers only	Interaction with dog and handler
Grubbs	2016	USA	Journal article	Dog	Exercise group	Exercise group with dogs and animal-assisted team
Gebhart	2020	Austria	Journal article	Dog	Distraction-focused interventions	Animal-assisted intervention with therapy dogs
Hansen	1999	USA	Journal article	Dog	Usual pediatric exam without a dog present	Pediatric examination in the presence of a dog
Hartfiel	2017	Germany	Journal article	Dog	Group therapy	Therapy session with animal
Hartwig	2017	USA	Journal article	Dog	Interactive and activity-based curriculum	Canine-assisted therapy based curriculum in HART intervention
Havener	2001	USA	Journal article	Dog	Dental procedure	Contact/interaction with a dog during dental procedure
Hediger	2019	Switzerland	Journal article	Horses, donkeys, sheep, goats, miniature pigs, cats, chickens, rabbits and guinea pigs	Conventional therapy session	Different therapies including an animal
Hediger	2019	Switzerland	Journal article	Dog, rabbits, guinea pigs	Occupational therapy	Animal-assisted therapy Affolter Concept
Henry	2015	USA	Journal article	Dog	Exercises involving focus on the body and physical movement	Intervention with dog
Hernandez-Espeso	2021	Spain	Journal article	Dolphin	Therapy without dolphins	Dolphin-assisted therapy and interaction with the therapist and the dolphin trainer
Hession	2019	Ireland	Journal article	Horses	Two control conditions: (a) audiovisual intervention or (b) waitlist	Horseback riding intervention
Heyer	2014	Germany	Journal article	Dog	Reading with plush dog	Reading with dog (active involvement of the dog)
Hinic	2019	USA	Journal article	Dog	Completed a jigsaw puzzle depicting an underwater scene with a research assistant and parent	Pet therapy with handler and dog, interaction with dog
Holman	2020	USA	Journal article	Dog	CBT manualized psychoeducational intervention	Canine-assisted therapy
Hunt	2014	USA	Journal article	Dog	Two control conditions: (a) write about a negative or traumatic event or (b) described in detail the dimensions and furnishings of three different rooms in three writing sessions	Writing in the presence of a dog
Hyeon Su	2014	South Korea	Journal article	Horse	Trunk stability exercise	Horseback riding
Janura	2015	Czech Republic	Journal article	Horse	Physiotherapy	Hippotherapy in addition to standard physiotherapy
Jasperson	2013	USA	Journal article	Dog	Group therapy	Intervention with dog
Johnson	2008	USA	Journal article	Dog	Two control conditions: (a) friendly human visit or (b) quiet reading group	Dog visit
Julius	2013	Germany	Journal article	Guinea pig	Empathy-training	Empathy-training with guinea pig
Kemeny	2021	USA	Journal article	Horse	HeartMath (HM) mindfulness-based intervention	Therapeutic horseback riding
Kim	2016	South Korea	Journal article	Horse	Horse riding simulator (HRS)	Horseback riding
Kim	2018	South Korea	Journal article	Horse	Simulated horseback riding	Horseback riding
Kim	2014	South Korea	Journal article	Horse	Treadmill Training	Horseback riding
Kline	2020	USA	Journal article	Dog	Coloring a mandala	Interaction with therapy dog
Ko	2016	South Korea	Journal article	Insects (crickets)	Lectures that focused on healthy lifestyle choices	Taking care of crickets
Kraft	2019	USA	Journal article	Horse	Standard outpatient physical therapy (PT)	Hippotherapy
Krause-Parello	2015	USA	Journal article	Dog	Standard forensic interview	AAI-canine in forensic interview
Krause-Parello	2019	USA	Journal article	Dog	Informational session about assistance dogs	Intervention with handler and therapy dog
Kwangmin Ryu	2016	South Korea	Journal article	Horse	Two control conditions: (a) aquatic movement therapy or (b) watching a movie	Horseback riding
Kwon	2015	South Korea	Journal article	Horse	Home-based aerobic exercise	Hippotherapy and active exercises
Lahav	2019	Israel	Journal article	Dog	Group intervention (solving problems and group sport)	Intervention with dog (educational topics about dog, getting to know the dog, interaction, practical training)
Lanning	2014	USA	Journal article	Horse	Educational and recreational activities	Equine-assisted activity to improve riding and horsemanship skills
Lang	2010	Germany	Journal article	Dog	A 30 min talk with the same research assistant	Dog-assisted interview
Lass-Hennemann	2018	Germany	Journal article	Dog	Two control conditions: (a) watching a 15-min film of a person interacting with one of the therapy dogs or (b) relaxing	Interaction with dog after traumatic film clip (physical contact was encouraged)
Lass-Hennemann	2014	Germany	Journal article	Dog	Three control conditions: (a) watching clip with friendly human, (b) watching clip with toy-animal or (c) watching clip alone	Interaction/ physical contact with dog during trauma film
LeRoux	2014	South Africa	Journal article	Dog	Tree control conditions: (a) reading to an adult, (b) reading to a teddy bear or (c) no intervention	Interacting with and reading out loud to dog
Lechner	2007	Switzerland	Journal article	Horse	Three control conditions: (a) sitting astride on Bobath Roll, (b) sitting on a rocker board (inside of a wooden stool) or (c) received no intervention	Horseback riding
Lee	2014	South Korea	Journal article	Horse	Treadmill	Hippotherapy
Lenihan	2016	USA	Journal article	Dog	Reading to adult volunteer	Weekly reading to same dog
Levinson	2017	USA	Journal article	Dog	Reading to peers	Reading to dog
Machova	2019	Czech Republic	Journal article	Dog	Standard physiotherapy and occupational therapy	Supplement of AAT
Machova	2018	Czech Republic	Journal article	Dog	Conventional speech therapy	Speech therapy with a dog
Machova	2020	Czech Republic	Journal article	Dog	Relaxation technique	AAA with a dog
Machova	2019	Czech Republic	Journal article	Dog	Two control conditions: (a) normal working process without a break or (b) normal working process with a break of choice	Work break in the presence of a dog
Marr	2000	USA	Journal article	Dogs, rabbits, ferrets, and guinea pigs	Substance abuse education group	Animal visit, free interaction with animal
Martos-Montes	2020	Spain	Journal article	Dog	Toy dog	Human-dog interaction
Matsuura	2020	Japan	Journal article	Horse	Stuffed toy horse	AAT with horse
Matusiak-Wieczorek	2020	Poland	Journal article	Horse	Less sessions of hippotherapy	Hippotherapy
Menna	2016	Italy	Journal article	Dog	Two control conditions: (a) activities based on the formal reality orientation (ROT) group or (b) no activities	AAT intervention with dog
Menna	2019	Italy	Journal article	Dog	Formal reality orientation (ROT) intervention without the dog	AAT with dog
Mossello	2011	Italy	Journal article	Dog	Control activity with plush dogs	Interaction with dog
Muela	2017	Spain	Journal article	Dog, horses; cats and farm animals (such as sheep, goats, chickens, and pigs)	Standard daily routine and psychotherapy	Treatment including animal (guided interactions with animals)
Mueller	2021	USA	Journal article	Dog	Two control conditions: (a) stuffed toy dog or (b) social interaction with animal	Social interaction and physical contact with a therapy dog
Munoz-Lasa	2011	Spain	Journal article	Horse	Physiotherapy	Horseback riding
Murry	2012	USA	Journal article	Reptile	The control group discussed death and grief without reference to, or interactions with, reptiles.	Reptile-assisted support group discussed death and grief along with training in animal care
Mutoh	2019	Japan	Journal article	Horse	Outdoor recreation program	Hippotherapy
Nathans-Barel	2005	Israel	Journal article	Dog	General discussions, learning about caring for animals, particularly dogs, and walks on hospital grounds with the therapist for similar periods as in the active group.	AAT with dog (interaction and activities)
Ngai	2021	Hong kong	Journal article	Dog	School program	Competence in Active Resilience for Kids (CARing Kids) humane education with animal- assisted SEL
Nilsson	2015	Sweden	Journal article	Dog	Visits only by researchers	Visits by researchers with an additional visit by a therapy dog and its handler.
Nurenberg	2015	USA	Journal article	Horses, dogs	Two control conditions: (a) environmentally enhanced social skills group psychotherapy (SSP) or (b) regular hospital care (standard control)	Equine-assisted-therapy
Odendaal	2001	USA	Disseration	dog	Read a book	Contact to dog (stroking)
O'Haire	2015	Australia	Journal article	Guinea pigs	Three control conditions: (a) playing with toys, (b) reading aloud or (c) reading silently	Freeplay with peers and animals
Oh	2018	South Korea	Journal article	Horse	Pharmacotherapy	Hippotherapy
Palsdottir	2020	Sweden	Journal article	Horse	Physical activity	Equine-assisted intervention
Pan	2019	USA	Journal article	Horse	Pony-sized stuffed horse, to practice activities (e.g., grooming and tacking)	Therapeutic horseback riding
Park	2019	South Korea	Journal article	Cricket	Auditory effects of pet crickets and telephone counseling	Insect-rearing
Pendry	2019	USA	Journal article	Dog	Academic Stress Management (ASM)	Interaction with therapy dog and handler or anti-stress management with dog
Pendry	2019a	USA	Journal article	Dog or cat	Two control conditions: (a) Watching others pet animal or (b) Viewing visuals of animals	Animal visitation program with dog or cat
Pendry	2020b	USA	Journal article	Dog	Academic stress management (ASM)	Interaction with therapy dog and handler or ASM with dog
Pendry	2021b	USA	Journal article	Dog	Academic stress management (ASM)	Interaction with therapy dog and handler or anti-stress management with dog
Pendry	2020	USA	Journal article	Dog	Academic stress management (ASM)	Interaction with therapy dog and handler or ASM with Dog
Pendry	2019a	USA	Journal article	Dog or cat	Two control conditions: (a) watching others pet animals or (b) viewing slideshow with animals	Animal visitation program with dog or cat
Peters	2021c	USA	Journal article	Horse	Occupational therapy in a garden	Equine-assisted therapy
Peters	2021c	USA	Journal article	Horse	Occupational therapy in a garden	Equine-assisted therapy
Petty	2017	USA	Journal article	Horse	Learning about horses	Horseback riding
Polheber	2014	USA	Journal article	Dog	Two control conditions: (a) speaking with their good friend or (b) sit quietly and wait	Interaction with dog
Rawleigh	2021	Canada	Journal article	Dog	Two control conditions: (a) a dog visitation program or (b) counseling	Dog training and vocational program
Richeson	2003	USA	Journal article	Dog	Two control conditions: (a) human visitor or (b) no visitors	Intervention with dog and handler
Rodrigo-Claverol	2019	Spain	Journal article	Dog	Kinesitherapy	Therapeutic exercises with animal
Rodrigo-Claverol	2020	Spain	Journal article	Dog	Physiotherapy	Physiotherapy + supplement of AAT
Ruzic	2011	Croatia	Journal article	Dog	Daily walk	Dog-walking
Santaniello	2020	Italy	Journal article	Dog	Formal reality orientation therapy (ROT)	AAT interventions adapted to the formal ROT
Scheidhacker	2002	Germany	Journal article	Horse	Horseback riding lesson	Therapeutic horseback riding
Schneider	2016	Canada	Journal article	Horse	Therapeutic skiing	Riding lessons
Schuck	2015	USA	Journal article	Dog	Two control conditions: (a) cognitive-behavioral intervention or (b) waitlist	Intervention with therapy dog and handler
Schuck	2018	USA	Journal article	Dog	Two control conditions: (a) cognitive-behavioral intervention with toy dog or (b) waitlist	Animal-assisted intervention with therapy dog and handler
Scorzato	2017	Italy	Journal article	Dog	Activity (substitution by an unanimated object)	Dog-assisted treatment intervention
Seivert	2014	USA	Journal article	Dog	Dog-walking	Dog training and education component
Smith	2010	USA	Dissertation	Dog	Read aloud independently in an assigned area of the public library	Reading sessions with therapy dog
Souza-Santos	2018	Brazil	Journal article	Horse	Dance	Horseback riding
Syzmanski	2018	USA	Journal article	Dog	Dog-walking	Training of undersocialized dogs
Temcharoensuk	2015	Thailand	Journal article	Horse	Two control conditions: (a) mechanical horse-riding simulator while watching an animated movie or (b) horse riding simulator was powered off	Horseback riding
Tepper	2021	Australia	Journal article	Dog	Two control conditions: (a) Dog present, (b) reading out lout to dog	Training with dog
Thakkar	2021	India	Journal article	Dog	Dental treatment	Dental treatment in the presence of a dog
Thelwell	2019	England	Journal article	Dog	Watching videos of dogs	10 min free interaction with dog
Thodberg	2016	Denmark	Journal article	Dog	Two control conditions: (a) interacting with a robot seal (PARO) or (b) interacting with a soft toy cat	Intervention with real life dog
Thodberg	2021	Denmark	Journal article	Dog	Two control conditions: (a) Visits with a dog, no activity (D) or (b) Visits without dog, with an activity (A).	Dog visit with activity
Travers	2013	Australia	Journal article	Dog	Human-therapist-only intervention with an article to stimulate discussion	Intervention with dog
Trujillo	2020	USA	Journal article	Dog	Manual-standardized motivational interviewing and acceptance and commitment therapy, called impACT	AAT + impACT
Urban	2015	Germany	Journal article	Dog	Walking with nurse	Dog walking
Vagnoli	2015	Italy	Journal article	Dog	Venipuncture without dog present	Venipuncture with dog present
Vandagriff	2021	USA	Journal article	Cats and dogs	Three control conditions: (a) animal visit program proximit,; (b) animal visit program imaginary or (c) waitlist	Free interaction with dog and cats, engaging in petting and stroking (for 10 min)
Spruin	2021	UK	Journal article	Cog	Mindfulness condition	Pets As Therapy (PAT) dog
Vidal Prieto	2021	Brasil	Journal article	Horse	Hippotherapy once a week	Hippotherapy twice a week
Villalta-Gil	2009	Spain	Journal article	Dog	Integrated psychological treatment	Intervention with dog
Voznesenskiy	2016	Ecuador	Journal article	Horses	Regular adapted physical education activities	Adaptive horseback riding
Wanser	2020	USA	Journal article	Dog	Dog walking intervention	“Do As I Do” dog training intervention
Wesenberg	2019	Germany	Journal article	Dog	Psychosocial group excercise sessions	Animal-assisted intervention
Wesley	2009	USA	Journal article	Dog	Group therapy session	Group therapy sessions with a therapy dog
White-Lewis	2019d	USA	Journal article	Horses	Evidence based exercise education	Equine-assisted therapy (grooming, saddling, riding)
White-Lewis	2018d	USA	Dissertation	Horses	Attention control exercise education group	Horseback riding
Wolynczyk-Gmaj	2021	Poland	Journal article	Dog	Walk with a researcher	Walk with dog and handler
Woolley	2004	USA	Dissertation	Dogs, cats, lamas, farm animals	Conventional psychotherapy only	Conventional psychotherapy and an AAT program
Zisselman	1996	USA	Journal article	Dog	Exercise control group	Dog visit

*AAA, animal-assisted activity; AAI, anima-assisted intervention; AAT, animal-assisted therapy*.

### Factor Hypotheses

**Figure 2:**
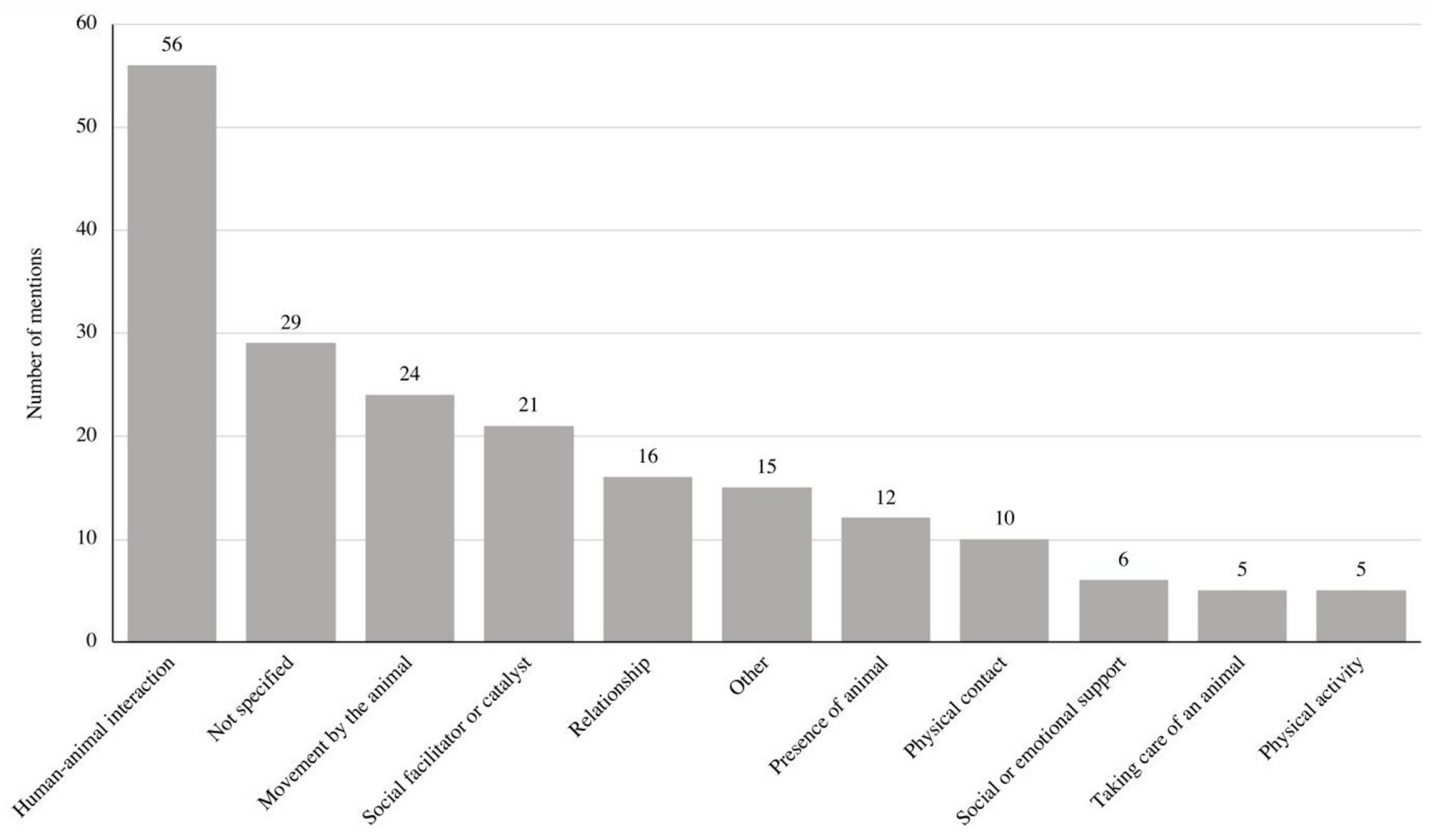
Number of identified factor hypotheses.

We defined the following eleven categories, sorted by frequency: (1) human–animal interaction, (2) not specified, (3) movement by the animal, (4) social facilitator or catalyst, (5) relationship with an animal, (6) other, (7) presence of an animal, (8) physical contact, (9) social or emotional support, (10) taking care of an animal, (11) physical activity (see Table 2; Figure 2). Detailed information about each factor-hypothesis category can be found in the Supplementary Material S2.

**Table 2:** Identified factor hypotheses, specific and non-specific factors of each study.

**Author**	**Year**	**Factor hypotheses**	**Specific factors**	**Non-specific factors**
Abdel-Aziem	2022	Movement by the animal	Animal; movement by the animal	Physical activity; therapeutic aspects
Alemdaroglu	2016	Not specified	Animal; movement by the animal	Physical activity
Allen	2021	Presence of animal	Animal	Therapeutic aspects; plush or toy animal
Ambrozy	2017	Movement by the animal	Animal, movement by the animal	Physical activity; environment
An	2021	Not specified	Animal; interaction with an animal	Physical activity; therapeutic aspects
Antonioli	2005	Other	Animal; interaction with an animal, taking care of an animal	Physical activity; environment; social contact
Aranda-Garcia	2015	Physical activity	Animal	Physical activity
Ashtari	2018	Relationship with an animal	Animal; interaction with an animal	Physical activity; environment; playing
Asqarova	2021	Not specified	Animal; interaction with an animal, social interaction	Activity, distraction, or absorption; education/training
Bachi	2014	Relationship	Animal	Education/training
Bailey	1987	Taking care of an animal	Animal; interaction with an animal	Social interaction; education or training
Banks	2008	Relationship with an animal	Animal	Social interaction; plush or toy animal
Barak	2001	Social facilitator/ or catalyst; relationship with an animal	Anima; taking care of an animal	Social interaction; activity, distraction, or absorption
Barker	2020	Not specified	Animal; interaction with an animal; social interaction	Activity, distraction, or absorption
Barker	2016	Human-animal interaction	Animal; interaction with an animal	Activity, distraction, or absorption
Barker	2003	Human-animal interaction	Animal; social interaction; interaction with an animal	Activity, distraction, or absorption
Beck	2012	Human-animal interaction	Animal; training an animal; physical contact; interaction with an animal	Therapeutic aspects
Becker	2017	Social facilitator or catalyst; human-animal interaction	Animal; physical contact; training an animal; taking care of an animal; social interaction	Social interaction; education or training
Beetz	2012	Social or emotional support	Animal; interaction with an animal; physical contact	Social interaction
Beetz	2015	Social facilitator or catalyst; physical contact	Animal; interaction with animal, movement by the animal	Therapeutic aspects; social interaction; activity, distraction, or absorption
Beinotti	2013	Movement by the animal; taking care of an animal; social facilitator or catalyst	Animal; physical contact	Physical activity
Beinotti	2010	Movement by the animal	Animal; movement by the animal	Physical activity
Benda	2003	Movement by the animal	Animal; movement by the animal	Activity, distraction, or absorption; interaction with something like an animal; relaxation; watching or seeing animal
Berget	2008	Taking care of an animal; human-animal interaction	Animal; physical contact; taking care of an animal	Therapeutic aspects
Berry	2012	Social facilitator or catalyst	Animal; interaction with an animal	Physical activity; therapeutic aspects; social interaction
Bialoszewski	2011	Human-animal interaction	Animal; movement by the animal	Physical activity; therapeutic aspects
Binfet	2022	Physical contact	Physical contact	Social interaction; animal
Bowin	2020	Human-animal interaction	Animal; interaction with an animal	Activity, distraction, or absorption
Boyer	2014	Social facilitator or catalyst	Animal	Plush or toy animal; interaction with something like an animal
Branson	2017	Human-animal interaction	Animal	Plush or toy animal; interaction with something like an animal; novelty
Bravo Gonçalves	2020	Other (mount material)	Other (mount material)	Animal; interaction with something like an animal; physical activity
Breitenbach	2009	Other (parental involvement)	Other (recreational/vacation atmosphere, therapeutic aspects)	Environment; animal; interaction with something like an animal
Bunketorp	2012	Movement by the animal	Animal; movement by the animal, taking care of an animal	Therapeutic aspects; movement or rhythm
Bunketorp	2019	Not specified	Animal; interaction with animal	Therapeutic aspects; social interaction; movement or rhythm
Calvo	2016	Social facilitator or catalyst; human-animal interaction	Animal; interaction with an animal; training an animal; taking care of an animal	Physical activity; therapeutic aspects; social interaction; activity, distraction, or absorption
Capparelli	2020	Social facilitator or catalyst; social or emotional support	Animal; interaction with an animal; physical contact	Activity, distraction, or absorption
Charnetski	2004	Physical contact; presence of animal	Animal; physical contact	Plush or toy animal; interaction with something like an animal; relaxation
Chen	2021	Human-animal interaction; social or emotional support	Animal; interaction with an animal	Therapeutic aspects; social interaction
Cho	2017	Movement by the animal	Animal	Physical activity; interaction with something like an animal; movement or rhythm
Clark	2020	Human-animal interaction	Animal; interaction with an animal	Social interaction
Cole	2007	Human-animal interaction; relationship with an animal	Animal; physical contact, interaction with an animal	Social interaction
Colombo	2006	Relationship with an animal	Animal	Other (taking care/responsibility)
Costa	2019	Human-animal interaction	Animal; interaction with an animal	Therapeutic aspects
Crossman	2015	Human-animal interaction	Animal; interaction with an animal	Watching or seeing animal
Crump	2015	Human-animal interaction; physical contact; relationship with an animal	Animal; interaction with an animal; physical contact	Activity, distraction, or absorption; social interaction
Dietz	2012	Not specified	Other (integrating dog in story)	Animal; social interaction; therapeutic aspects
Dunalp	2020	Presence of animal	Animal	Social interaction; education or training
Eckes	2020	Taking care of an animal	Animal; taking care of an animal	Social interaction; education or training
El-Maniawy	2012	Movement by the animal	Animal; movement by the animal	Physical activity
Fiocco	2017	Human-animal interaction	Animal; interaction with an animal	Relaxation
Flynn	2019	Social facilitator or catalyst	Animal; interaction with an animal; taking care of an animal	Education or training
Foerder	2021	Human-animal interaction	Animal, interaction with an animal	Plush or toy animal; social interaction
Friedmann	2015	Social facilitator or catalyst; social or emotional support	Animal	Therapeutic aspects; social interaction; activity, distraction, or absorption
Funakoshi	2018	Movement by the animal	Animal	Physical activity, movement or rhythm, interaction with something like an animal
Fung	2014	Social facilitator or catalyst	Animal	Therapeutic aspects; social interaction; activity, distraction, or absorption
Gabriels	2015	Human-animal interaction; relationship with an animal	Animal; movement by the animal; taking care of an animal, interaction with an animal	Plush ortoy animal; education or training; therapeutic aspects; environment
Gabriels	2018	Human-animal interaction; relationship with an animal	Animal; movement by the animal; taking care ofan animal; interaction with an animal	Plush or toy animal; education or training; therapeutic aspects; environment
Germone	2019	Social facilitator or catalyst	Animal; interaction with an animal	Social interaction; activity, distraction, or absorption; novelty
Gocheva	2018	Not specified	Animal; interaction with an animal; taking care of an animal	Therapeutic aspects; physical activity; activity, distraction, or absorption
Gee	2019	Other (biophilia)	Animal; other (distraction presence of animal)	Environment; activity, distraction, or absorption
Grajforner	2017	Human-animal interaction	Interaction with an animal; social interaction	Social interaction; animal
Grubbs	2016	Social facilitator or catalyst	Animal; interaction with an animal; social interaction	Physical activity; social interaction
Gebhart	2020	Not specified	Animal; interaction with an animal	Movement or rhythm; activity, distraction, or absorption; social interaction; other (distraction)
Hansen	1999	Presence of animal; other (distraction)	Animal; interaction with an animal	Therapeutic aspects
Hartfiel	2017	Social facilitator or catalyst	Animal	Social interaction
Hartwig	2017	Not specified	Animal	Therapeutic aspects; social interaction
Havener	2001	Relationship with an animal; other (distraction)	Animal; interaction with an animal	Therapeutic aspects
Hediger	2019	Animal as social facilitator or catalyst	Animal	Therapeutic aspects
Hediger	2019	Not specified	Animal	Therapeutic aspects
Henry	2015	Human-animal interaction	Animal; interaction with an animal	Physical activity; therapeutic aspects; activity, distraction, or absorption
Hernandez-Espeso	2021	Human-animal interaction	Animal; interaction with an animal	Environment; therapeutic aspects; social interaction
Hession	2019	Movement by the animal	Animal; movement by the animal	Therapeutic aspects; movement or rhythm; watching or seeing animal
Heyer	2014	Other (integrating real-life animal)	Animal; interaction with an animal	Plush or toy animal; activity, distraction, or absorption
Hinic	2019	Not specified	Animal; interaction with an animal	Social interaction; activity, distraction, or absorption; education or training
Holman	2020	Human-animal interaction	Animal, interaction with an animal; physical contact; social interaction	Therapeutic aspects
Hunt	2014	Social facilitator or catalyst	Animal	Activity, distraction, or absorption
Hyeon Su	2014	Movement by the animal	Animal; movement by the animal	Physical activity
Janura	2015	Movement by the animal	Animal; movement by the animal	Therapeutic aspects; physical activity
Jasperson	2013	Human-animal interaction	Animal; interaction with an animal; physical contact	Therapeutic aspects; social interaction; education
Johnson	2008	Not specified	Animal; interaction with an animal; taking care of an animal; physical contact	Social interaction; activity, distraction, or absorption
Julius	2013	Human-animal interaction	Animal	Therapeutic aspects; education or training
Kemeny	2021	Human-animal interaction; other (large animal)	Animal; interaction with an animal; movement by the animal; relationship with an animal	Therapeutic aspects; relaxation
Kim	2016	Not specified	Animal	Physical activity; movement or rhythm; interaction with something like an animal
Kim	2018	Physical activity	Animal	Physical activity; movement or rhythm; interaction with something like an animal
Kim	2014	Movement by the animal	Animal; movement by the animal	Physical activity
Kline	2020	Human-animal interaction	Animal; interaction with an animal	Activity, distraction, or absorption
Ko	2016	Human-animal interaction	Animal; taking care of an animal	Social interaction; education or training
Kraft	2019	Movement by the animal	Animal; movement by the animal	Therapeutic aspects; physical activity
Krause-Parello	2015	Not specified	Animal; physical contact	Activity, distraction, or absorption
Krause-Parello	2019	Not specified	Animal; interaction with an animal	Education or training
Kwangmin Ryu	2016	Movement by the animal	Animal; movement by the animal	Physical activity; environment; activity, distraction, or absorption
Kwon	2015	Movement by the animal	Animal; movement by the animal	Physical activity; therapeutic aspects
Lahav	2019	Social facilitator or catalyst; presence of animal	Animal; training an animal; interaction with an animal; other (educational topics of animal);	Physical activity; social interaction, activity, distraction, or absorption
Lanning	2014	Movement by the animal; relationship with an animal	Animal; movement by the animal; taking care of an animal	Social interaction; activity, distraction, or absorption; education or training
Lang	2010	Not specified	Animal; interaction with an animal	Social interaction; other (talking about pet/animals)
Lass-Hennemann	2018	Human-animal interaction	Animal; interaction with an animal; physical contact	Watching or seeing animal; animal; interaction with something like an animal
Lass-Hennemann	2014	Social or emotional support; presence of animal	Animal; interaction with an animal; physical contact	Plush or toy animal; social interaction; activity, distraction, or absorption
LeRoux	2014	Not specified	Animal	Plush or toy animal; social interaction; activity, distraction, or absorption
Lechner	2007	Movement by the animal	Animal; movement by the animal	Physical activity
Lee	2014	Movement by the animal	Animal; movement by the animal	Physical activity
Lenihan	2016	Human-animal interaction	Animal; relationship with the animal	Social interaction; activity, distraction, or absorption
Levinson	2017	Not specified	Animal	Social interaction; activity, distraction, or absorption
Machova	2019	Presence of animal	Animal; interaction with an animal; relationship with an animal	Therapeutic aspects; physical activity
Machova	2018	Presence of animal	Animal; interaction with an animal; physical contact	Therapeutic aspects
Machova	2020	Human-animal interaction	Animal; interaction with an animal	Relaxation
Machova	2019	Presence of animal	Animal; interaction with an animal	Relaxation
Marr	2000	Social facilitator or catalyst	Animal; interaction with an animal	Social interaction; education or training
Martos-Montes	2020	Human-animal interaction	Animal; interaction with an animal	Plush or toy animal
Matsuura	2020	Physical contact	Physical contact	Plush or toy animal; interaction with something like an animal; watching or seeing animal
Matusiak-Wieczorek	2020	Not specified	Other (frequency)	Animal; movement or rhythm
Menna	2016	Relationship with an animal	Animal; interaction with an animal	Therapeutic aspects
Menna	2019	Human-animal interaction; physical contact	Animal; interaction with an animal	Therapeutic aspects
Mossello	2011	Physical contact	Animal	Plush or toy animal; interaction with something like an animal
Muela	2017	Not specified	Animal	Therapeutic aspects
Mueller	2021	Human-animal interaction; physical contact	Physical contact; interaction with an animal	Plush or toy animal; novelty
Munoz-Lasa	2011	Movement by the animal	Animal; movement by the animal	Physical activity; therapeutic aspects
Murry	2012	Taking care of an animal	Animal; taking care of an animal; other (education about animal)	Social interaction; education or training
Mutoh	2019	Movement by the animal	Animal; movement by the animal	Environment; activity, distraction, or absorption
Nathans-Barel	2005	Human-animal interaction	Animal; interaction with an animal	Physical activity; social interaction, education or training
Ngai	2021	Not specified	Animal; interaction with an animal	Education or training
Nilsson	2015	Human-animal interaction	Animal; interaction with an animal; physical contact	Social interaction
Nurenberg	2015	Not specified	Animal; interaction with an animal; training animal	Therapeutic aspects; environment; social interaction
Odendaal	2001	Human-animal interaction	Other (familiarity)	Activity, distraction, or absorption
O'Haire	2015	Social facilitator or catalyst	Animal; interaction with an animal	Social interaction; activity, distraction, or absorption
Oh	2018	Human-animal interaction	Animal; movement by the animal	Therapeutic aspects
Palsdottir	2020	Not specified	Animal; movement by the animal; social interaction	Physical activity
Pan	2019	Human-animal interaction	Animal; movement by the animal	Plush or toy animal; activity, distraction, or absorption; interaction with something like an animal
Park	2019	Other (animal can create nostalgia feeling)	Animal; taking care of an animal	Other (sound of animal); therapeutic aspects
Pendry	2019	Human-animal interaction	Animal; interaction with an animal	Therapeutic aspects; physical activity, education or training
Pendry	2019a	Human-animal interaction	Interaction with an animal; social interaction	Activity, distraction, or absorption; watching or seeing animal; other (proximity)
Pendry	2020b	Human-animal interaction	Animal; interaction with an animal	Therapeutic aspects; activity, distraction, or absorption; education or training
Pendry	2021b	Human-animal interaction	Animal; interaction with an animal	Therapeutic aspects; activity, distraction, or absorption; education or training
Pendry	2020	Human-animal interaction	Animal; interaction with an animal	Therapeutic aspects; activity, distraction, or absorption; education or training
Pendry	2019a	Human-animal interaction	Interaction with an animal; social interaction	Activity, distraction, or absorption; watching or seeing animal; other (proximity)
Peters	2021c	Not specified	Animal; interaction with an animal	Therapeutic aspects; environment, social interaction; education or training; activity, distraction, or absorption
Peters	2021c	Human-animal interaction, social facilitator or catalyst	Animal; interaction with an animal	Therapeutic aspects; environment; social interaction, education or training; activity, distraction, or absorption
Petty	2017	Human-animal interaction; relationship with an animal	Animal; movement by the animal; taking care of an animal	Education or training; environment; plush ortoy animal
Polheber	2014	Social or emotional support	Animal; interaction with an animal	Social interaction; relaxation
Rawleigh	2021	Human-animal interaction	Training an animal	Animal; therapeutic aspects
Richeson	2003	Relationship with an animal	Animal; interaction with an animal	Social interaction; activity, distraction, or absorption
Rodrigo-Claverol	2019	Human-animal interaction	Animal	Physical activity; therapeutic aspects
Rodrigo-Claverol	2020	Not specified	Animal; interaction with an animal; physical contact	Therapeutic aspects; physical activity; social interaction
Ruzic	2011	Physical activity	Animal; taking care of an animal	Physical activity
Santaniello	2020	Human-animal interaction	Animal; interaction with an animal	Therapeutic aspects
Scheidhacker	2002	Not specified	Other (therapeutic aspects)	Animal; other (horseback riding)
Schneider	2016	Human-animal interaction	Animal; movement by the animal	Physical activity; therapeutic aspects
Schuck	2015	Social facilitator or catalyst; human-animal interaction	Animal	Therapeutic aspects; plush or toy animal
Schuck	2018	Human-animal interaction	Animal	Therapeutic aspects; plush or toy animal
Scorzato	2017	Not specified	Animal; interaction with an animal	Activity, distraction, or absorption
Seivert	2014	Human-animal interaction	Training an animal; relationship with an animal	Animal; physical activity
Smith	2010	Not specified	Animal	Activity, distraction, or absorption; environment
Souza-Santos	2018	Physical activity	Animal; physical contact; movement by the animal	Physical activity; social interaction
Syzmanski	2018	Human-animal interaction	Training an animal	Animal; physical activity
Temcharoensuk	2015	Movement by the animal	Animal	Physical activity; activity, distraction, or absorption; interaction with something like an animal
Tepper	2021	Human-animal interaction	Training an animal	Animal
Thakkar	2021	Physical contact	Animal	Therapeutic aspects
Thelwell	2019	Human-animal interaction	Animal, interaction with an animal	Watching or seeing animal
Thodberg	2016	Human-animal interaction	Animal	Plush or toy animal; interaction with something like an animal
Thodberg	2021	Presence of animal	Other (combination of activity with dog)	Animal; physical contact; activity, distraction, or absorption
Travers	2013	Social facilitator or catalyst; physical contact	Animal; interaction with an animal	Social interaction; other (bringing article to stimulate discussion)
Trujillo	2020	Social facilitator or catalyst	Animal	Therapeutic aspects
Urban	2015	Not specified	Animal	Physical activity; social interaction; environment
Vagnoli	2015	Not specified	Animal	Therapeutic aspects
Vandagriff	2021	Physical contact	Physical contact; interaction with an animal	Animal; watching or seeing animal; other (proximity)
Spruin	2021	Not specified	Animal; interaction with an animal	Activity, distraction, or absorption; relaxation
Vidal Prieto	2021	Movement by the animal	Other (frequency)	Animal; movement or rhythm
Villalta-Gil	2009	Not specified	Animal; interaction with an animal	Therapeutic aspects; social interaction
Voznesenskiy	2016	Physical activity	Animal; movement by the animal	Physical activity
Wanser	2020	Relationship with an animal	Training an animal	Animal; physical activity
Wesenberg	2019	Presence of animal	Animal; physical contact; taking care of an animal; training of an animal	Physical activity; social interaction
Wesley	2009	Social facilitator or catalyst	Animal	Therapeutic aspects; social interaction
White-Lewis d)	2019	Movement by the animal	Animal; movement by the animal	Physical activity; education or training
White-Lewis d)	2018	Movement by the animal	Animal; movement by the animal, taking care of an animal; training an animal	Physical activity; education or training
Wolynczyk-Gmaj	2021	Presence of animal	Animal	Physical activity; social interaction
Woolley	2004	Not specified	Animal, interaction with an animal; taking care of an animal	Therapeutic aspects; social interaction
Zisselman	1996	Relationship with an animal	Animal; interaction with an animal; social interaction	Physical activity

#### Human–Animal Interaction

This category subsumed hypotheses that held the positive impact of human–animal interaction in general as responsible for the effects of AAIs. For example, authors stated that the interaction with an animal can reduce human stress (e.g., Barker et al., 2016; Fiocco and Hunse, 2017) or anxiety (e.g., Crossman et al., 2015; Foerder and Royer, 2021) or increase oxytocin levels (e.g., Chen et al., 2021). We found that 32.56% (*n* = 56) of the analyzed studies hypothesized human–animal interaction to be the working mechanism of AAIs.

#### Not Specified

This category contained studies where the authors did not specify possible mechanisms, made general assumptions, or mentioned different mechanisms in their introduction without specifying in the end what they hypothesized to be the working mechanism. For example, if authors mentioned that AAIs can lead to stress relief but did not specify what leads to this stress relief (such as interacting with the animal), the hypothesis was categorized as not specified (e.g., Gocheva et al., 2018; Bunketorp-Kall et al., 2019; An and Park, 2021). The results show that 16.86% (*n* = 29) of the studies did not specify factor hypotheses.

#### Movement by the Animal

In this category, we subsumed hypotheses that assumed that movement by the animal is crucial for the effects of AAIs. This includes, for example, the movement or rhythm of a horse when riding (e.g., Ambrozy et al., 2017; Hession et al., 2019; Kraft et al., 2019). We found that authors of 24 studies mentioned movement as a mechanism for the effects of AAIs, which accounted for 13.95% of the analyzed studies.

#### Social Facilitator or Catalyst

In this category, we included studies that hypothesized that animals' ability to act as social facilitators or catalysts has positive effects on humans. For example, authors hypothesized that animals enhance social learning in humans (Schuck et al., 2015) or foster human social communication and interaction skills (e.g., Barak et al., 2001; Flynn et al., 2019). The analyses revealed that 12.21% (*n* = 21) of the analyzed studies mentioned the animal as a social facilitator or catalyst as a possible mechanism for the effects of AAIs.

#### Relationship With an Animal

In this category, we subsumed hypotheses addressing the positive effect of relationships, attachment, or companionship between humans and animals. For example, some authors mentioned the positive effect of an attachment (e.g., Crump and Derting, 2015) or relationship established over time between a patient and an animal (Lanning et al., 2014). The results show that 16 studies mentioned the relationship between humans and animals as an explanation for the mechanisms of AAIs. This accounted for 9.3% of the analyzed studies.

#### Other

In this category, we summarized hypotheses that were not mentioned more than twice and did not match any other category. Examples include the biophilia hypothesis (e.g., Antonioli and Reveley, 2005; Gee et al., 2019) or the hypothesis that the sound of insects can create nostalgic feelings (Park et al., 2019). In total, we identified 15 studies with other factor hypotheses, which accounted for 8.72% of the analyzed studies.

#### Presence of Animal

In this category we included all studies that considered the presence of an animal as a possible mechanism of AAIs. For example, some claimed that the presence of an animal (in contrast to interacting with an animal) has a calming effect (Allen et al., 2021) or can distract from stressful situations (Hansen et al., 1999). We found that 6.98% (*n* = 12) of the studies mentioned the presence of an animal as a possible mechanism.

#### Physical Contact

This category encompassed hypotheses addressing physical contact with the animal as a possible mechanism of AAIs. For example, some authors suggested that petting an animal increases autonomic arousal (Vandagriff et al., 2021). We found that 10 studies mentioned physical contact as a possible mechanism of AAIs, which accounted for 5.81% of the analyzed studies.

#### Social or Emotional Support

In this category, we included hypotheses that animals can provide either social or emotional support to humans. An example is the suggestion that an animal can provide social support comparable to that of a human (Lass-Hennemann et al., 2014). Authors of six studies mentioned animals as social or emotional support as a hypothesis for the effects of AAIs. This accounted for 3.49% of the analyzed studies.

#### Taking Care of an Animal

In this category, we included studies where the authors hypothesized that the opportunity to take care of an animal can enhance the effects of AAIs (e.g., Murry and Allen, 2012; Eckes et al., 2020). We found five studies where authors mentioned this as a potential mechanism of AAIs. This accounted for 2.91% of the analyzed studies.

#### Physical Activity

We subsumed hypotheses about the importance of physical activity for the effects of AAIs in this category. For example, some authors suggested that exercising with animals (e.g., walking with an animal) leads to an effect (Aranda-Garcia et al., 2015). In total, 2.91% (*n* = 5) of the analyzed studies mentioned physical activity as a possible mechanism of AAIs.

### Specific Factors of AAIs

**Figure 3:**
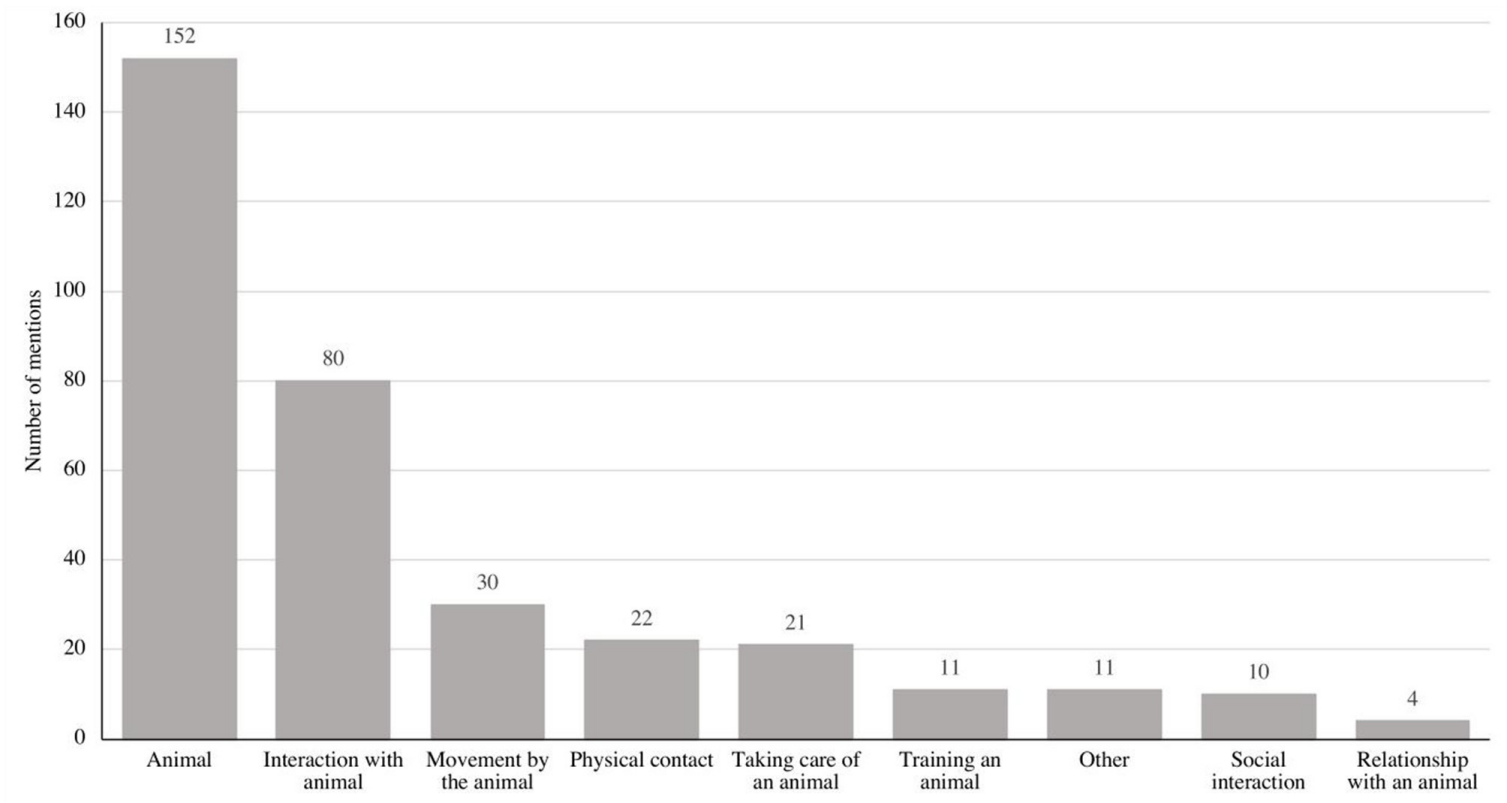
Number of identified specific factors.

We identified nine categories of specific factors of AAIs that were reflected in the control conditions of published AAI studies. Ordered by frequency, these categories were: (1) animal, (2) interaction with an animal, (3) movement by the animal, (4) physical contact, (5) taking care of an animal, (6) training an animal, (7) other, (8) social interaction, (9) relationship with an animal (see Table 2; Figure 3). A detailed description of all the categories of specific factors can be found in the Supplementary Material S3.

#### Animal

In the category “animal,” we included studies that had an experimental condition with a live animal and that compared that condition to a control condition with no animal present (e.g., Julius et al., 2013; Kim et al., 2016; Branson et al., 2017; Hartfiel et al., 2017; Levinson et al., 2017; Schuck et al., 2018; Wolynczyk-Gmaj et al., 2021; Abdel-Aziem et al., 2022). We found that 88.37% (*n* = 152) of the studies controlled for an animal as a specific factor.

#### Interaction With an Animal

Here we included studies with experimental conditions that contained a specific form of interaction with an animal, such as playing with an animal or free interaction (e.g., Hansen et al., 1999; Machova et al., 2019; Gebhart et al., 2020). We also included petting in this category if it was only mentioned as one of many ways that subjects could interact with an animal (e.g., Crump and Derting, 2015; Gocheva et al., 2018). If physical contact was part of the intervention—for example, if participants had to pet an animal—we categorized the factor under “physical contact” (e.g., Charnetski et al., 2004; Binfet et al., 2022). Further, in this category, we included studies that defined the reaction of the animal—such as sounds or other responses—as important for the interaction. Analyses revealed that 46.51% (*n* = 80) of the studies controlled for the interaction with an animal as a specific factor.

#### Movement by the Animal

In this category, we included studies with experimental conditions that incorporated movement by an animal as part of the intervention, such as while horseback riding (e.g., Lechner et al., 2007; Kim et al., 2014; Alemdaroglu et al., 2016; Abdel-Aziem et al., 2022). We determined that 17.44% (*n* = 30) of the studies controlled for movement as a specific factor.

#### Physical Contact

In this category, we included studies with experimental conditions that specified physical contact with an animal, such as petting, as the factor in their intervention (e.g., Crump and Derting, 2015; Holman et al., 2020; Binfet et al., 2022). We found that 12.79% (*n* = 22) of the studies controlled for physical contact as a specific factor.

#### Taking Care of an Animal

Here, we included studies with experimental conditions where participants took care of an animal, for example, by grooming, feeding, or milking it (e.g., Berget and Braastad, 2008; Ko et al., 2016; Gocheva et al., 2018). Of the analyzed studies, 12.21% (*n* = 21) defined taking care of an animal as a specific factor.

#### Training an Animal

In this category, we included studies with experimental conditions where subjects could teach or train animals, for example, by giving animal commands (e.g., Rawleigh and Purc-Stephenson, 2021). We found that 11 studies included training animals as a specific factor, which accounted for 6.39% of the analyzed studies.

#### Other

Here we included studies with characteristics in their experimental conditions that did not match any other category and that were not mentioned more than twice. Examples in this category are mounting material (Bravo Gonçalves Junior et al., 2020), the familiarity of the animal (Odendaal, 2001), or the frequency of the intervention (Vidal Prieto et al., 2021). We found 11 studies that controlled for other specific factors. This accounted for 6.39% of the included studies.

#### Social Interaction

In this category, we included studies with experimental conditions where subjects engaged with other human beings, for example, in group activities or by talking to another person (e.g., Palsdottir et al., 2020; Asqarova, 2020). Analyses showed that 5.81% (*n* = 10) of the studies controlled for social interaction as a specific factor.

#### Relationship With an Animal

In this category, we included studies with experimental conditions where relationship-building between subjects and an animal was promoted, for example, when subjects could work for a longer time with one animal in order to build a relationship with the animal (e.g., Seivert, 2014). We found that 2.32% (*n* = 4) of the studies controlled for the relationship with the animal as a specific factor.

### Non-specific Factors of AAIs

**Figure 4:**
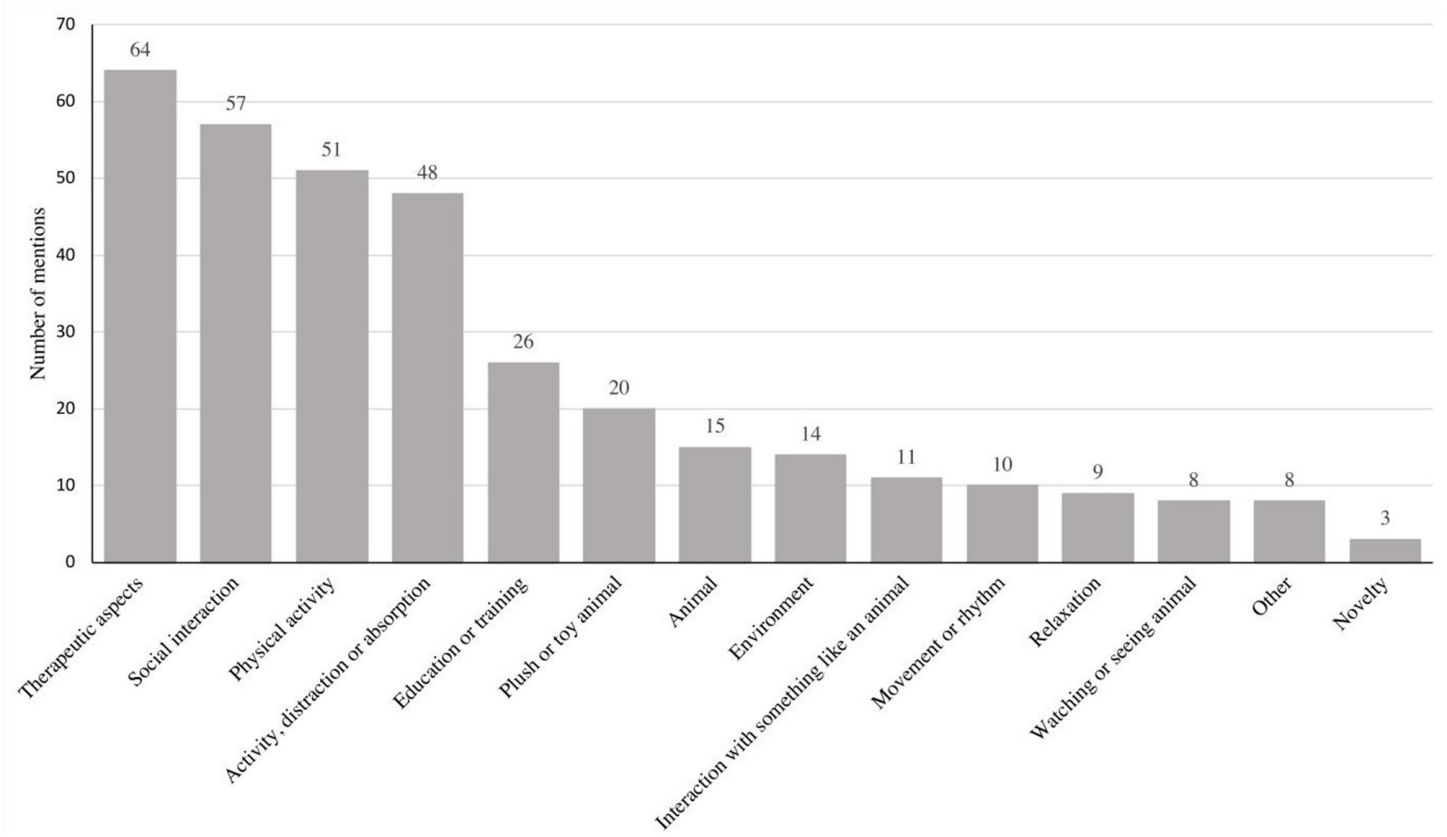
Number of identified non-specific factors.

Comparing the control and the experimental condition in previously published studies, we identified the following 14 categories of non-specific factors, ordered by frequency: (1) therapeutic aspects, (2) social interaction, (3) physical activity, (4) activity, distraction, or absorption, (5) education or training, (6) plush or toy animal, (7) animal, (8) environment, (9) interaction with something like an animal, (10) movement or rhythm, 11) relaxation, (12) watching or seeing an animal, (13) other, and (14) novelty (see Table 2; Figure 4). Detailed information about the non-specific categories can be found in the Supplementary Material S4.

#### Therapeutic Aspects

In this category, we included studies with control conditions that had a therapeutic component, such as trauma-focused therapy (e.g., Allen et al., 2021), psychological treatment (e.g., Muela et al., 2017; Holman et al., 2020), or physiotherapeutic treatment (e.g., Beinotti et al., 2013; Rodrigo-Claverol et al., 2020). In total, 37.21% (*n* = 64) of the analyzed studies controlled for therapeutic aspects as a non-specific factor.

#### Social Interaction

Here we included studies with control conditions that contained contact or interaction with other humans, such as speaking to another human or playing group sports (e.g., Crump and Derting, 2015; Grubbs et al., 2016; Foerder and Royer, 2021). Analyses showed that 57 studies controlled for social contact or interaction as a non-specific factor. This accounted for 33.14% of the included studies.

#### Physical Activity

In this category, we included studies with control conditions that controlled for physical activity, such as rehabilitation exercises (e.g., Alemdaroglu et al., 2016), group sports (e.g., Calvo et al., 2016), or dance classes (e.g., Souza-Santos et al., 2018). We found that 51 studies controlled for physical activity as a non-specific factor. This accounted for 29.65% of the included studies.

#### Activity, Distraction, or Absorption

In this category, we subsumed studies with control conditions that offered an activity or that distracted or occupied participants or demanded their attention by, for example, having them read (e.g., Heyer and Beetz, 2014; Barker et al., 2020), color (e.g., Kline et al., 2020), or write (e.g., Hunt and Chizkov, 2014). Of the analyzed studies, 27.91% (*n* = 48) controlled for activity, distraction, or absorption as a non-specific factor.

#### Education or Training

Here we included studies with control conditions that contained educational aspects, such as social-skills training (e.g., Becker et al., 2017) or empathy training (e.g., Julius et al., 2013; Dunlap, 2020). We found that 15.17% (*n* = 26) of the studies controlled for education or training as a non-specific factor.

#### Plush or Toy Animal

In this category, we included all studies with control interventions that incorporated a plush or toy animal, such as a plush dog (e.g., Branson et al., 2017), toy dog (e.g., Martos-Montes et al., 2020), or stuffed plush horse (e.g., Gabriels et al., 2018). We found that 20 studies controlled for interacting with a plush or toy animal as a non-specific factor. This accounted for 11.63% of the included studies.

#### Animal

In this category, we included studies with control conditions where subjects had contact with a live animal but where the degree of contact and interaction varied. For example, in one study, the animal in the control condition was only present (compared to training with the animal in the experimental condition) (Tepper et al., 2021), or some studies compared control conditions in which subjects interacted with an animal, such as by walking with a dog, to working with an animal in the experimental condition (Seivert, 2014). We found that 15 studies controlled for the presence, contact, or interaction with the animal as a non-specific factor. This accounted for 8.72% of the included studies.

#### Environment

In this category, we included studies that controlled for environmental factors, such as being in water (e.g., Antonioli and Reveley, 2005; Hernandez-Espeso et al., 2021), being outdoors (e.g., Urban et al., 2015), or being on a farm (e.g., Breitenbach et al., 2009) in the control condition. We found that 14 studies controlled for the environment as a non-specific factor. This accounted for 8.14% of the included studies.

#### Interaction With Something Like an Animal

In this category, we included studies with control conditions that simulated human–animal interaction or contact with another object by, for example, grooming a plush cat (e.g., Boyer and Mundschenk, 2014) or riding a mechanical horse (e.g., Kim et al., 2016; Funakoshi et al., 2018). We found that 11 studies controlled for interaction with something like an animal as a non-specific factor. This accounted for 6.35% of the included studies.

#### Movement or Rhythm

All studies with conditions that controlled for movement or rhythm were included in this category. They included rhythm and music-based therapy (e.g., Bunketorp Kall et al., 2012) or the vibrations or movements of a mechanic horse (Cho, 2017; Funakoshi et al., 2018; Kim et al., 2018). We found that 5.81% (*n* = 10) of the studies controlled for rhythm or movement as a non-specific factor.

#### Relaxation

In this category, we included studies with control conditions where subjects were asked to sit and relax for a certain amount of time (Fiocco and Hunse, 2017; Machová et al., 2020a,b). We found that nine studies controlled for relaxation as a non-specific factor. This accounted for 5.23% of the included studies.

#### Watching or Seeing Animal

Here we included studies with control conditions that exposed subjects to visual stimuli of animals, such as through videos or pictures (e.g., Hession et al., 2019; Thelwell, 2019; Vandagriff et al., 2021). We found eight studies that controlled for watching or seeing an animal as a non-specific factor. This accounted for 4.65% of the included studies.

#### Other

In this category, we included studies with characteristics of the control condition that did not match any other category, such as the sound of an animal (Park et al., 2019) or a proximity effect (Vandagriff et al., 2021). We found that 4.65% (*n* = 8) of the studies controlled for other factors as non-specific factors.

#### Novelty

In this category, we included studies that controlled for a novelty effect by including control conditions with novel toys or plush animals (Branson et al., 2017; Germone et al., 2019; Mueller et al., 2021). We found three studies that controlled for a novelty effect as non-specific factor. This accounted for 1.74% of the included studies.

## Discussion

The aim of this systematic review was to present an overview of explicit factor hypotheses that researchers have presented in previous AAI studies and to identify factors that have been implicitly considered as specific factors or non-specific factors in AAI research.

### Factor Hypotheses of AAIs

We found that the majority of the studies (84%) mentioned a hypothesis about how AAI works. However, a substantial portion (16%) of the analyzed studies did not specify any factor hypotheses referring to concrete working mechanisms of AAIs in their introductions. The most frequently mentioned factor hypothesis was that human–animal interaction leads to the effects of AAIs, followed by movement by the animals, animals as social facilitators or catalysts, and the presence of an animal. These extracted factor hypotheses all represent hypothesized working mechanisms by the authors, but most of them are not sufficiently specific for authors to avoid making assumptions about how different specific components of AAIs contribute to its effects. While human–animal interaction was mentioned by several authors as a specific factor, human–animal interaction comprises a multitude of components. For example, several studies hypothesized that human–animal interaction can reduce stress (Fiocco and Hunse, 2017; Pan et al., 2019; Machová et al., 2020b), but they did not specify how human–animal interaction leads to this possible stress-reducing effect. These rather vague factor hypotheses about human–animal interaction and AAIs reflect the current problem in the AAI research where the question of *how* AAIs work is still neglected (López-Cepero, 2020).

Nevertheless, our review also revealed that some studies defined factor hypotheses that are quite specific, such as the movement of the involved animals. For example, the tridimensional (Cho, 2017; Vidal Prieto et al., 2021), repetitive (Funakoshi et al., 2018; Vidal Prieto et al., 2021), and rhythmic movements of a horse (Vidal Prieto et al., 2021) have been defined as specific factors of horseback riding that are assumed to have positive effects on the humans riding the horse. But given the strong and decade-old recommendations in the literature to specify what characteristics of AAIs are important for the effects (Marino, 2012; López-Cepero, 2020), we were surprised not to find more specific factor hypotheses. We strongly suggest that authors explicitly state their hypotheses about how the presence of an animal may enhance interventions.

### Specific Factors of AAIs

Based on the approach of component studies, which provide a method for examining the active components of a treatment, we compared the control conditions with the experimental conditions of each study. We defined a factor as specific if it was present in the experimental condition but not in the control condition. We identified that “animal” and “interaction with an animal” were the most frequent categories that previously published AAI studies have implicitly considered a specific and active component of AAIs. By using different control conditions, the studies also controlled for specific factors such as “movement by the animal,” “physical contact,” and “taking care of an animal.” For example, “movement by the animal” was controlled for by comparing horseback riding with physiotherapy (e.g., Abdel-Aziem et al., 2022), “physical contact” by comparing being interviewed while petting a dog to being interviewed without a dog (Krause-Parello and Gulick, 2015), and “taking care of an animal” by comparing participants attending lectures about healthy lifestyle choices with participants taking care of crickets (Ko et al., 2016).

The results indicate that the authors of the majority of studies implicitly considered the animal as a specific factor of the AAI. This reflects the common assumption in the AAI literature that the animal is crucial for the effects of AAIs (Marino, 2012). However, since the animal is itself a complex stimulus (Marino, 2012; Rodriguez et al., 2021) and since interaction with an animal has many different components, the animal might not be suitable as a specific factor. But the results make clear what steps are needed in AAI research. First, studies need to investigate if the animal is a specific factor and if it is needed for the effects of AAIs. And then the effects of different characteristics of animals need to be disentangled.

One characteristic of an animal that we found defined as a specific factor in several on studies equine-assisted interventions (17%) was the movement of a horse during riding. Especially in hippotherapy, research is already investigating highly specific mechanisms. If the movement of a horse is considered a specific factor in equine-assisted interventions, the question arises if this movement needs to be performed by a live horse or if it can be substituted. Similar questions are increasingly being addressed, for example, in this specific case by comparing the effects of riding on a real horse with riding on a horse stimulator (Temcharoensuk et al., 2015; Kim et al., 2016, 2018; Cho, 2017).

Although rarely mentioned, we also identified factors that were considered as specific but were independent of the animal, such as mounting material (Bravo Gonçalves Junior et al., 2020), distraction by the presence of an animal (Gee et al., 2019), frequency of the intervention (Matusiak-Wieczorek et al., 2020), familiarity with the animal (Odendaal, 2001), recreational aspects (Breitenbach et al., 2009), and therapeutic aspects (Scheidhacker et al., 2002; Breitenbach et al., 2009). This indicates that researchers are beginning to investigate and to understand what factors in AAIs can be separated from the animal.

### Non-specific Factors of AAIs

We found that previous AAI studies have already controlled for several different non-specific factors. We considered a factor to be implicitly defined as non-specific if it was present in both the experimental and the control intervention. Most frequently, therapeutic aspects and social interactions were identified as non-specific factors. For example, some studies compared a control condition consisting of standard physiotherapy while the experimental condition consisted of standard physiotherapy with the addition of an animal (Berry et al., 2012; Machova et al., 2019; Rodrigo-Claverol et al., 2020). We thus interpreted the authors of these studies to be attempting to control for non-specific effects of the therapeutic context present in both interventions.

Some of the studies also controlled for specific elements of the interaction with the animal or the animal itself, for example, by defining the presence of an animal (Tepper et al., 2021) or simply walking with a dog (Syzmanski et al., 2018) as non-specific factors. One such study had a control group with an animal present during classroom activities and an experimental group where participants interacted with an animal to complete different tasks (Tepper et al., 2021). Another study defined walking with a dog as the control intervention, while the experimental intervention had participants train dogs to be more suitable for adoption (Syzmanski et al., 2018). Other examples of such specific factors of an animal were the sound of an animal (Park et al., 2019), proximity to an animal (Pendry and Vandagriff, 2019; Pendry et al., 2019; Vandagriff et al., 2021), or taking care of another living being (Colombo et al., 2006). We also found that a minority of studies defined novelty as a non-specific factor. While only Mueller et al. (2021) explicitly mentioned having a stuffed toy present in the control group to control for the novelty effect of the animal in the intervention group, we interpreted two other studies also to be controlling for novelty when they included “novel” toys in the control condition (Branson et al., 2017; Germone et al., 2019). It has already been suggested that AAIs might be prone to novelty effects, which is thus a threat to construct validity (Marino, 2012), so it is rather surprising that we only identified one study that specifically controlled for novelty as a non-specific effect. This also makes clear how important it is for authors to explicitly mention their hypotheses about working mechanisms and what they considered in designing the control and the experimental conditions. Having a stuffed toy present can function as a control for different components such as feeling fur, being confronted with a novel stimulus, or receiving support.

Moreover, AAIs are thought to be vulnerable to placebo effects because the nature of the treatment is usually evident to the subjects (Marino, 2012). Studies on placebo effects have demonstrated that psychosocial and contextual factors related to patient perceptions of the intervention—including information about the treatment, expectations, and the treatment environment—can contribute to the overall effect of the intervention (Wager and Atlas, 2015). Moreover, research has shown that a significant part of our responses to various interventions can be explained by these contextual factors and thus by mechanisms that elicit placebo effects rather than by the specific intervention itself (Wager and Atlas, 2015). In randomized controlled trials, such contextual factors are usually controlled for with a placebo control (Colloca and Benedetti, 2005). The results from our systematic review show, however, that none of the included studies explicitly controlled for placebo effects. Dietz et al. (2012) investigated the effects of animal-assisted therapy on trauma symptoms and compared animal-assisted therapy not only to a control group but also had an intervention group that was provided narratives about the therapy dog while the other intervention group received no such narratives about the dog. Such stories might have influenced the expectations of the participants, but the authors did not mention that these conditions were intended to control for participants' expectations as a part of a placebo effect. The lack of a control for placebo effects in previous AAI research may have led to false attributions: it might not be the animal that produces the effects of AAIs but rather participants' expectations regarding the animal or a combination of both. Considering that a large part of treatment responses in other interventions such as psychotherapy or physiotherapy (Wampold, 2015; Testa and Rossettini, 2016) can be explained by contextual factors rather than by their specific factors, it seems likely that these factors also explain a large portion of the effects in AAIs.

### Limitations, Strengths, and Future Research

Several studies we analyzed lacked detailed information regarding the study design and the experimental and the control conditions. Since we identified factors by looking at the study design and by comparing the control and experimental conditions, the information about the way the animal was integrated in the intervention was crucial for our results. For example, it was sometimes not clear if the animal was just present or embedded in a therapeutic narrative, what role the animal had, what amount of physical contact occurred, or even if participants rode the horses they were working with. This lack of information could have affected our categories and whether they correctly reflect the studies. For example, we might have missed specific or non-specific factors that were taken into account. We also included only English and German publications and were not able to obtain several manuscripts. Moreover, our categories reflect a subjective classification. Finally, we only analyzed studies with active control conditions. Authors of studies without a control group might have proposed hypotheses about working mechanisms that we thus missed. A strength of this review is that we included previously published controlled studies with different types of AAIs. We thus ensured that the results are representative of different fields ranging from dog-assisted interventions to hippotherapy to educational programs including animals. In order to minimize publication bias, we also included non-peer-reviewed manuscripts, though the study quality was sometimes low. Our review presents a representative overview of the current status of hypotheses about specific and non-specific factors in AAI research based both on explicit statements by authors and on implicit measures. This is a significant step in addressing a crucial knowledge gap and provides a basis for recommendations for future research.

In future studies, authors should clearly state their hypotheses about the working mechanisms. As López-Cepero (2020) suggested, integrating an animal in human services should be justified through mechanisms that we can hypothesize and that then can be verified through a scientific methodology.

Similar to other treatments like psychotherapy, AAIs are faced with the challenge of identifying how and why AAIs lead to changes (Kazdin, 2007, 2009). In order to understand how AAIs work, identifying specific factors in AAIs is crucial. We propose using component studies to examine the active components of AAIs. This means that future studies need to carefully plan their control conditions. The results of this review provide some indications of how the familiarity of the animal (Odendaal, 2001) or the relationship to the animal (Seivert, 2014; Machova, 2019) could be considered as specific factors to be controlled for, but further specific factors should be identified. Moreover, future research should try to disentangle the specific effects by treating the animal as a complex stimulus. Authors should try to define and examine exactly what characteristics are specific to the animal and what characteristics can be substituted by a human or a non-living animal. By using robotic dogs, for example, certain confounding components such as novelty, demand characteristics, expectations, caring for someone, and physical activity can be controlled for. To design good component studies on AAIs, we hypothesize that future studies need more specific and innovative control interventions. We recommend that future studies not only examine more specifically which components of the animal or of the interaction with the animal may have effects but also start to acknowledge and implement knowledge from placebo research to examine the impact of contextual factors in AAIs. We believe that this will help us better understand the mechanisms of AAIs and also determine how important the animal is for the effects of AAIs. The results of this review show that some non-specific factors such as therapeutic aspects and social interaction have already been controlled for in past studies, which suggests that the field is moving in the right direction. However, we suggest that future research pays attention to patients' perceptions of the intervention such as information and expectations about the treatment, the treatment environment, and the therapeutic alliance. It could even be argued that the animal in AAIs may not need to be a specific factor but could rather be seen as a contextual factor. We hope to stimulate this debate in future research with this paper.

## Conclusion

A substantial portion of previously published controlled AAI studies did not define specific hypotheses about working mechanisms. By analyzing their control conditions, we assumed that in most controlled studies, the animal or the interaction with an animal were implicitly considered a specific factor for the effects of AAIs. Non-specific factors such as therapeutic aspects, social interaction, or novelty have also been controlled for. We conclude that AAI research still cannot answer the question of how and why AAIs work. The hypotheses and results about the specific and non-specific factors in the literature on AAIs are insufficient. This poses a major knowledge gap and challenge for the future. With this paper, we have presented the first overview of what AAI research has considered as possible specific and non-specific factors. These can be used in future research to address the question of the mechanisms of AAIs. To disentangle the mechanisms of AAIs, future research should employ component studies with innovative control conditions and draw on knowledge from placebo research.

## Data Availability Statement

The raw data supporting the conclusions of this article will be made available by the authors, without undue reservation.

## Author Contributions

CW and KH had the idea for the study and designed the study. CW and CG contributed to acquiring the data and carried out the analysis. CW, KH, and CG wrote the manuscript, which was revised by all authors. All authors contributed to the article and approved the submitted version.

## Funding

KH received support from an Eccellenza Professorial Fellowship from the Swiss National Science Foundation (Grant PCEFP1_194591).

## Conflict of Interest

The authors declare that the research was conducted in the absence of any commercial or financial relationships that could be construed as a potential conflict of interest.

## Publisher's Note

All claims expressed in this article are solely those of the authors and do not necessarily represent those of their affiliated organizations, or those of the publisher, the editors and the reviewers. Any product that may be evaluated in this article, or claim that may be made by its manufacturer, is not guaranteed or endorsed by the publisher.
