# Supplementary material for: Specific and Non-specific Factors of Animal-Assisted Interventions Considered in Research: A Systematic Review
Source: Front Psychol. 2022 Jun 28;13:931347. doi: 10.3389/fpsyg.2022.931347 (PMC9274084; doi:10.3389/fpsyg.2022.931347)
Supplement: Supplementary file 1 [file Data_Sheet_1.docx]

**SUPPLEMENTARY MATERIALS**

**Table of Contents**

S1: Methods: Search strings

S2: Results: Detailed information of the categories of the factor hypotheses

S3: Results: Detailed information of the categories of the specific-factors

S4: Results: Detailed information of the categories of the nonspecific factors

**SI: Methods: Search strings**

**PsycINFO (Ovid)**

(("Animal intervention*" or "Animal therap*" or "Animal assisted" or "Animal facilitated" or Anthrozoology or "Assistance animal*" or
"Assistance dog*" or "Assistance horse*" or "Canine therap*" or "Canine assisted" or "Canine facilitated" or "Companion animal*" or

"Dog therap*" or "Dog assisted" or "Dog facilitated" or "Dolphin therap*" or "Dolphin assisted" or "Dolphin facilitated" or "Equine therap*" or "Equine assisted" or "Equine facilitated" or Hippotherapy or "Horseback riding" or "Human animal bond*" or
"Human animal interaction*" or "Pet therap*" or "Pet assisted" or "Pet facilitated" or "Service animal*" or "Service dog*" or
"Service horse*" or "Therapeutic animal*" or "Therapeutic dog*" or "Therapeutic horse*" or "Therapeutic pet*" or "Therapeutic riding" or
"Therapy with animal*").ti,ab,id OR ("Animal Assisted Therapy" OR "Service animals").sh or ("Animal Assisted Therapy" or "Bonding, Human-Pet"
or "Equine-Assisted Therapy").mh) and ((Interspecies or animal* or pet* or "human animal*" or dog* or canine* or equine* or horse* or dolphin* or mammal* or goat* or cat* or pig*
or rabbit* or bird* or sheep or chicken* or turtle* or fish or aquarium or farm*).ti,ab,id. OR (Mammals OR "Interspecies Interaction" OR Dogs OR
Animals OR Pets OR Horses OR Dolphins OR Goats OR Cats OR "Guinea Pigs" OR Pigs OR Rabbits OR Birds OR Sheep OR Chickens OR Turtles OR Fishes).sh
or (Animals or "Animals, Domestic" or Pets or Dogs or Cats or Sheep or Horses or Dolphins or Mammals or Goats or "Guinea Pigs" or Swine or Rabbits
or Birds or Chickens or Turtles or Fishes or Farms).mh) and (("Controlled trial*" or "Control group*").ti,ab,id. or ("Randomized Controlled Trials").sh
or ("Randomized Controlled Trial").mh)

**PSYNDEX (OVID)**

(("Animal intervention*" or "Animal therap*" or "Animal assisted" or "Animal facilitated" or Anthrozoology or "Assistance animal*" or
"Assistance dog*" or "Assistance horse*" or "Canine therap*" or "Canine assisted" or "Canine facilitated" or "Companion animal*" or

"Dog therap*" or "Dog assisted" or "Dog facilitated" or "Dolphin therap*" or "Dolphin assisted" or "Dolphin facilitated" or
"Equine therap*" or "Equine assisted" or "Equine facilitated" or Hippotherapy or "Horseback riding" or "Human animal bond*" or

"Human animal interaction*" or "Pet therap*" or "Pet assisted" or "Pet facilitated" or "Service animal*" or "Service dog*" or
"Service horse*" or "Therapeutic animal*" or "Therapeutic dog*" or "Therapeutic horse*" or "Therapeutic pet*" or "Therapeutic riding" or

"Therapy with animal*").ti,ab,id OR ("Animal Assisted Therapy" OR "Service animals").sh) and ((Interspecies or animal* or pet* or "human animal*" or dog* or canine* or equine* or horse* or dolphin* or mammal* or goat* or cat* or pig* or rabbit* or bird* or sheep or chicken* or turtle* or fish or aquarium or farm*).ti,ab,id. OR (Mammals OR "Interspecies Interaction" OR Dogs OR Animals OR Pets OR Horses OR Dolphins OR Goats OR Cats OR "Guinea Pigs" OR Pigs OR Rabbits OR Birds OR Sheep OR Chickens OR Turtles OR Fishes).sh) and

(("Controlled trial*" or "Control group*").ti,ab,id. or ("Randomized Controlled Trials").sh)

**ERIC (Ovid)**

(("Animal intervention*" or "Animal therap*" or "Animal assisted" or "Animal facilitated" or Anthrozoology or "Assistance animal*" or
"Assistance dog*" or "Assistance horse*" or "Canine therap*" or "Canine assisted" or "Canine facilitated" or "Companion animal*" or

"Dog therap*" or "Dog assisted" or "Dog facilitated" or "Dolphin therap*" or "Dolphin assisted" or "Dolphin facilitated" or
"Equine therap*" or "Equine assisted" or "Equine facilitated" or Hippotherapy or "Horseback riding" or "Human animal bond*" or

"Human animal interaction*" or "Pet therap*" or "Pet assisted" or "Pet facilitated" or "Service animal*" or "Service dog*" or
"Service horse*" or "Therapeutic animal*" or "Therapeutic dog*" or "Therapeutic horse*" or "Therapeutic pet*" or "Therapeutic riding" or

"Therapy with animal*").ti,ab,id or (Animals and Therapy).sh) and ((Interspecies or animal* or pet* or "human animal*" or dog* or canine* or equine* or horse* or dolphin* or mammal* or goat* or cat* or pig*
or rabbit* or bird* or sheep or chicken* or turtle* or fish or aquarium or farm*).ti,ab,id. OR (Animals).sh) and (("Controlled trial*" or "Control group*").ti,ab,id. or ("Randomized Controlled Trials").sh)

**MEDLINE (Ovid)**

(("Animal intervention*" or "Animal therap*" or "Animal assisted" or "Animal facilitated" or Anthrozoology or "Assistance animal*" or
"Assistance dog*" or "Assistance horse*" or "Canine therap*" or "Canine assisted" or "Canine facilitated" or "Companion animal*" or

"Dog therap*" or "Dog assisted" or "Dog facilitated" or "Dolphin therap*" or "Dolphin assisted" or "Dolphin facilitated" or
"Equine therap*" or "Equine assisted" or "Equine facilitated" or Hippotherapy or "Horseback riding" or "Human animal bond*" or

"Human animal interaction*" or "Pet therap*" or "Pet assisted" or "Pet facilitated" or "Service animal*" or "Service dog*" or
"Service horse*" or "Therapeutic animal*" or "Therapeutic dog*" or "Therapeutic horse*" or "Therapeutic pet*" or "Therapeutic riding" or

"Therapy with animal*").ti,ab,id or ("Animal Assisted Therapy" or "Bonding, Human-Pet"
or "Equine-Assisted Therapy").sh) and ((Interspecies or animal* or pet* or "human animal*" or dog* or canine* or equine* or horse* or dolphin* or mammal* or goat* or cat* or pig*
or rabbit* or bird* or sheep or chicken* or turtle* or fish or aquarium or farm*).ti,ab,id. OR (Animals or "Animals, Domestic" or Pets or Dogs
or Cats or Sheep or Horses or Dolphins or Mammals or Goats or "Guinea Pigs" or Swine or Rabbits or Birds or Chickens or Turtles or Fishes or Farms).sh) and (("Controlled trial*" or "Control group*").ti,ab,id. or ("Randomized Controlled Trial").sh)

**Embase (Ovid)**

(("Animal intervention*" or "Animal therap*" or "Animal assisted" or "Animal facilitated" or Anthrozoology or "Assistance animal*" or
"Assistance dog*" or "Assistance horse*" or "Canine therap*" or "Canine assisted" or "Canine facilitated" or

"Dog therap*" or "Dog assisted" or "Dog facilitated" or "Dolphin therap*" or "Dolphin assisted" or "Dolphin facilitated" or
"Equine therap*" or "Equine assisted" or "Equine facilitated" or Hippotherapy or "Horseback riding" or "Human animal bond*" or

"Human animal interaction*" or "Pet therap*" or "Pet assisted" or "Pet facilitated" or "Service animal*" or "Service dog*" or
"Service horse*" or "Therapeutic animal*" or "Therapeutic dog*" or "Therapeutic horse*" or "Therapeutic pet*" or "Therapeutic riding" or

"Therapy with animal*").ti,ab,kw or ("animal assisted therapy" or "human-animal bond" or "service dog" or hippotherapy).sh) and ((Interspecies or animal* or pet* or "human animal*" or dog* or canine* or equine* or horse* or dolphin* or mammal* or goat* or cat* or pig* or rabbit* or bird* or sheep or chicken* or turtle* or fish or aquarium or farm*).ti,ab,kw. or (animal or "PET ANIMAL" or DOG or CANIDAE

or HORSE or "toothed whale" or MAMMAL or GOAT or CAT or "GUINEA PIG" or PIG or Leporidae or BIRD or SHEEP or CHICKEN or TURTLE or FISH or rabbit or "agricultural land" or "domestic pig" or "domestic animal").sh) and
(("Controlled trial*" or "Control group*").ti,ab,kw. or ("controlled study").sh) and

(psychiatry).ec

**PubMed**

("Animal intervention"[Title/Abstract] or "Animal therapy"[Title/Abstract] or "Animal assisted"[Title/Abstract] or "Animal facilitated"[Title/Abstract]
or Anthrozoology[Title/Abstract] or "Assistance animal"[Title/Abstract] or "Assistance dog"[Title/Abstract] or "Assistance horse"[Title/Abstract]

or "Canine therapy"[Title/Abstract] or "Canine assisted"[Title/Abstract] or "Canine facilitated"[Title/Abstract] or "Companion animal"[Title/Abstract] or
"Dog therapy"[Title/Abstract] or "Dog assisted"[Title/Abstract] or "Dog facilitated"[Title/Abstract] or "Dolphin therapy"[Title/Abstract] or
"Dolphin assisted"[Title/Abstract] or "Dolphin facilitated"[Title/Abstract] or "Equine therapy"[Title/Abstract] or "Equine assisted"[Title/Abstract]
or "Equine facilitated"[Title/Abstract] or Hippotherapy[Title/Abstract] or "Horseback riding"[Title/Abstract] or "Human animal bond"[Title/Abstract]
or "Human animal interaction"[Title/Abstract] or "Pet therapy"[Title/Abstract] or "Pet assisted"[Title/Abstract] or "Pet facilitated"[Title/Abstract]
or "Service animal"[Title/Abstract] or "Service dog"[Title/Abstract] or "Service horse"[Title/Abstract] or "Therapeutic animal"[Title/Abstract]
or "Therapeutic dog"[Title/Abstract] or "Therapeutic horse"[Title/Abstract] or "Therapeutic pet*"[Title/Abstract] or "Therapeutic riding"[Title/Abstract]
or "Therapy with animals" [Title/Abstract] or "Animal Assisted Therapy"[MeSH Terms] or "Bonding, Human-Pet"[MeSH Terms] or "Equine-Assisted Therapy"[MeSH Terms]) and (Interspecies[Title/Abstract] or animal[Title/Abstract] or pet[Title/Abstract] or "human animal"[Title/Abstract] or dog[Title/Abstract] or canine[Title/Abstract]
or equine[Title/Abstract] or horse[Title/Abstract] or dolphin[Title/Abstract] or mammal[Title/Abstract] or goat[Title/Abstract] or cat[Title/Abstract] or pig[Title/Abstract]
or rabbit[Title/Abstract] or bird[Title/Abstract] or sheep[Title/Abstract] or chicken[Title/Abstract] or turtle[Title/Abstract] or fish[Title/Abstract] or aquarium[Title/Abstract]
or farm[Title/Abstract] or Animals[MeSH Terms] or "Animals, Domestic"[MeSH Terms] or Pets[MeSH Terms] or Dogs[MeSH Terms] or Cats[MeSH Terms] or Sheep[MeSH Terms]
or Horses[MeSH Terms] or Dolphins[MeSH Terms] or Mammals[MeSH Terms] or Goats[MeSH Terms] or "Guinea Pigs"[MeSH Terms] or Swine[MeSH Terms] or Rabbits[MeSH Terms]
or Birds[MeSH Terms] or Chickens[MeSH Terms] or Turtles[MeSH Terms] or Fishes[MeSH Terms] or Farms[MeSH Terms]) and ("Controlled trial"[Title/Abstract] or "Control group"[Title/Abstract] or "Randomized Controlled Trial"[MeSH Terms])

**Cochrane Library**

("Animal intervention*" or "Animal therap*" or "Animal assisted" or "Animal facilitated" or Anthrozoology or "Assistance animal*" or "Assistance dog*" or
"Assistance horse*" or "Canine therap*" or "Canine assisted" or "Canine facilitated" or "Companion animal*" or "Dog therap*" or "Dog assisted" or "Dog facilitated"

or "Dolphin therap*" or "Dolphin assisted" or "Dolphin facilitated" or "Equine therap*" or "Equine assisted" or "Equine facilitated" or Hippotherapy
or "Horseback riding" or "Human animal bond*" or "Human animal interaction*" or "Pet therap*" or "Pet assisted" or "Pet facilitated" or "Service animal*"

or "Service dog*" or "Service horse*" or "Therapeutic animal*" or "Therapeutic dog*" or "Therapeutic horse*" or "Therapeutic pet*" or "Therapeutic riding"
or "Therapy with animal*") AND (Interspecies or animal* or pet* or "human animal*" or dog* or canine* or equine* or horse* or dolphin* or mammal* or goat*
or cat* or pig* or rabbit* or bird* or sheep or chicken* or turtle* or fish or aquarium or farm*) AND ("Controlled trial*" or "Control group*")

**Web of Science**

(("Animal intervention*" or "Animal therap*" or "Animal assisted" or "Animal facilitated" or Anthrozoology or "Assistance animal*" or
"Assistance dog*" or "Assistance horse*" or "Canine therap*" or "Canine assisted" or "Canine facilitated" or "Companion animal*" or

"Dog therap*" or "Dog assisted" or "Dog facilitated" or "Dolphin therap*" or "Dolphin assisted" or "Dolphin facilitated" or
"Equine therap*" or "Equine assisted" or "Equine facilitated" or Hippotherapy or "Horseback riding" or "Human animal bond*" or

"Human animal interaction*" or "Pet therap*" or "Pet assisted" or "Pet facilitated" or "Service animal*" or "Service dog*" or
"Service horse*" or "Therapeutic animal*" or "Therapeutic dog*" or "Therapeutic horse*" or "Therapeutic pet*" or "Therapeutic riding" or

"Therapy with animal*") and (Interspecies or animal* or pet* or "human animal*" or dog* or canine* or equine* or horse* or dolphin* or mammal* or goat* or cat* or pig*
or rabbit* or bird* or sheep or chicken* or turtle* or fish or aquarium or farm*) and ("Controlled trial*" or "Control group*"))

**Scopus**

(TITLE-ABS-KEY ( "Animal intervention*" or "Animal assisted" or "Animal facilitated" or Anthrozoology or "Assistance animal*" or
"Assistance dog*" or "Assistance horse*" or "Canine therap*" or "Canine assisted" or "Canine facilitated" or "Dog therap*" or "Dog assisted" or

"Dog facilitated" or "Dolphin therap*" or "Dolphin assisted" or "Dolphin facilitated" or "Equine therap*" or "Equine assisted" or "Equine facilitated"
or Hippotherapy or "Horseback riding" or "Human animal bond*" or "Human animal interaction*" or "Pet therap*" or "Pet assisted" or "Pet facilitated"
or "Service animal*" or "Service dog*" or "Service horse*" or "Therapeutic animal*" or "Therapeutic dog*" or "Therapeutic horse*" or "Therapeutic pet*"
or "Therapeutic riding" or "Therapy with animal*" ) AND ( "Controlled trial*" or "Control group*" ) )

**CINAHL (EBSCO)**

(TI ("Animal intervention*" or "Animal therap*" or "Animal assisted" or "Animal facilitated" or Anthrozoology or "Assistance animal*" or
"Assistance dog*" or "Assistance horse*" or "Canine therap*" or "Canine assisted" or "Canine facilitated" or "Companion animal*" or

"Dog therap*" or "Dog assisted" or "Dog facilitated" or "Dolphin therap*" or "Dolphin assisted" or "Dolphin facilitated" or
"Equine therap*" or "Equine assisted" or "Equine facilitated" or Hippotherapy or "Horseback riding" or "Human animal bond*" or

"Human animal interaction*" or "Pet therap*" or "Pet assisted" or "Pet facilitated" or "Service animal*" or "Service dog*" or
"Service horse*" or "Therapeutic animal*" or "Therapeutic dog*" or "Therapeutic horse*" or "Therapeutic pet*" or "Therapeutic riding" or

"Therapy with animal*") OR AB ("Animal intervention*" or "Animal therap*" or "Animal assisted" or "Animal facilitated" or Anthrozoology or "Assistance animal*" or
"Assistance dog*" or "Assistance horse*" or "Canine therap*" or "Canine assisted" or "Canine facilitated" or "Companion animal*" or

"Dog therap*" or "Dog assisted" or "Dog facilitated" or "Dolphin therap*" or "Dolphin assisted" or "Dolphin facilitated" or
"Equine therap*" or "Equine assisted" or "Equine facilitated" or Hippotherapy or "Horseback riding" or "Human animal bond*" or

"Human animal interaction*" or "Pet therap*" or "Pet assisted" or "Pet facilitated" or "Service animal*" or "Service dog*" or
"Service horse*" or "Therapeutic animal*" or "Therapeutic dog*" or "Therapeutic horse*" or "Therapeutic pet*" or "Therapeutic riding" or

"Therapy with animal*") OR SU ("Animal Assisted Therapy (Iowa NIC)" or "Service Animals" or "Equine-Assisted Therapy" or "Human-Pet Bonding" or "Pet Therapy"))
AND (TI (Interspecies or animal* or pet* or "human animal*" or dog* or canine* or equine* or horse* or dolphin* or mammal* or goat* or cat* or pig*

or rabbit* or bird* or sheep or chicken* or turtle* or fish or aquarium or farm*) OR AB (Interspecies or animal* or pet* or "human animal*" or dog* or canine*
or equine* or horse* or dolphin* or mammal* or goat* or cat* or pig* or rabbit* or bird* or sheep or chicken* or turtle* or fish or aquarium or farm*)

OR SU (animals OR pets OR dogs OR horses OR DOLPHINS OR mammals OR goats OR cats OR "guinea pigs" OR swine OR rabbits OR birds OR sheep OR turtles OR fish))
AND (TI ("Controlled trial*" or "Control group*") OR AB ("Controlled trial*" or "Control group*") OR SU ("Randomized Controlled Trials"))

**PTSDpubs (ProQuest)**

[STRICT](TI( "Animal intervention*" or "Animal therap*" or "Animal assisted" or "Animal facilitated" or Anthrozoology or "Assistance animal*" or "Assistance dog*" or
"Assistance horse*" or "Canine therap*" or "Canine assisted" or "Canine facilitated" or "Companion animal*" or "Dog therap*" or "Dog assisted" or "Dog facilitated" or

"Dolphin therap*" or "Dolphin assisted" or "Dolphin facilitated" or "Equine therap*" or "Equine assisted" or "Equine facilitated" or Hippotherapy or "Horseback riding"
or "Human animal bond*" or "Human animal interaction*" or "Pet therap*" or "Pet assisted" or "Pet facilitated" or "Service animal*" or "Service dog*" or "Service horse*"
or "Therapeutic animal*" or "Therapeutic dog*" or "Therapeutic horse*" or "Therapeutic pet*" or "Therapeutic riding" or "Therapy with animal*" ) OR AB ( "Animal intervention*"
or "Animal therap*" or "Animal assisted" or "Animal facilitated" or Anthrozoology or "Assistance animal*" or "Assistance dog*" or "Assistance horse*" or "Canine therap*" or
"Canine assisted" or "Canine facilitated" or "Companion animal*" or "Dog therap*" or "Dog assisted" or "Dog facilitated" or "Dolphin therap*" or "Dolphin assisted" or
"Dolphin facilitated" or "Equine therap*" or "Equine assisted" or "Equine facilitated" or Hippotherapy or "Horseback riding" or "Human animal bond*" or "Human animal interaction*"
or "Pet therap*" or "Pet assisted" or "Pet facilitated" or "Service animal*" or "Service dog*" or "Service horse*" or "Therapeutic animal*" or "Therapeutic dog*"

or "Therapeutic horse*" or "Therapeutic pet*" or "Therapeutic riding" or "Therapy with animal*" ) OR SU( "Animal Assisted Therapy" )) AND (TI( Interspecies or animal* or pet* or "human animal*" or dog* or canine* or equine* or horse* or dolphin* or mammal* or goat* or cat* or pig*
or rabbit* or bird* or sheep or chicken* or turtle* or fish or aquarium or farm* ) OR AB ( Interspecies or animal* or pet* or "human animal*" or dog* or canine*
or equine* or horse* or dolphin* or mammal* or goat* or cat* or pig* or rabbit* or bird* or sheep or chicken* or turtle* or fish or aquarium or farm* ) OR
SU ( pets OR animals OR dogs OR horses OR "dolphins & porpoises" OR mammals OR "marine mammals" OR cats OR rabbits OR birds OR sheep OR fish OR aquariums OR farms )) AND (TI( "Controlled trial*" or "Control group*" ) OR AB( "Controlled trial*" or "Control group*" ) OR SU( "Randomized Clinical Trial" ))

**Dissertations & Theses (ProQuest)**

[STRICT](TI( "Animal intervention*" or "Animal therap*" or "Animal assisted" or "Animal facilitated" or Anthrozoology or "Assistance animal*" or "Assistance dog*" or
"Assistance horse*" or "Canine therap*" or "Canine assisted" or "Canine facilitated" or "Companion animal*" or "Dog therap*" or "Dog assisted" or "Dog facilitated" or

"Dolphin therap*" or "Dolphin assisted" or "Dolphin facilitated" or "Equine therap*" or "Equine assisted" or "Equine facilitated" or Hippotherapy or "Horseback riding"
or "Human animal bond*" or "Human animal interaction*" or "Pet therap*" or "Pet assisted" or "Pet facilitated" or "Service animal*" or "Service dog*" or "Service horse*"
or "Therapeutic animal*" or "Therapeutic dog*" or "Therapeutic horse*" or "Therapeutic pet*" or "Therapeutic riding" or "Therapy with animal*" ) OR AB ( "Animal intervention*"
or "Animal therap*" or "Animal assisted" or "Animal facilitated" or Anthrozoology or "Assistance animal*" or "Assistance dog*" or "Assistance horse*" or "Canine therap*" or
"Canine assisted" or "Canine facilitated" or "Companion animal*" or "Dog therap*" or "Dog assisted" or "Dog facilitated" or "Dolphin therap*" or "Dolphin assisted" or
"Dolphin facilitated" or "Equine therap*" or "Equine assisted" or "Equine facilitated" or Hippotherapy or "Horseback riding" or "Human animal bond*" or "Human animal interaction*"
or "Pet therap*" or "Pet assisted" or "Pet facilitated" or "Service animal*" or "Service dog*" or "Service horse*" or "Therapeutic animal*" or "Therapeutic dog*"
or "Therapeutic horse*" or "Therapeutic pet*" or "Therapeutic riding" or "Therapy with animal*" ) OR SU( "animal assisted therapy" )) AND (TI( Interspecies or animal* or pet* or "human animal*" or dog* or canine* or equine* or horse* or dolphin* or mammal* or goat* or cat* or pig*
or rabbit* or bird* or sheep or chicken* or turtle* or fish or aquarium or farm* ) OR AB ( Interspecies or animal* or pet* or "human animal*" or dog* or canine*
or equine* or horse* or dolphin* or mammal* or goat* or cat* or pig* or rabbit* or bird* or sheep or chicken* or turtle* or fish or aquarium or farm* ) OR
SU ( pets OR animals OR dogs OR horses OR "dolphins & porpoises" OR mammals OR "marine mammals" OR cats OR rabbits OR birds OR sheep OR fish OR aquariums OR farms )) AND (TI("Controlled trial*" or "Control group*") OR AB( "Controlled trial*" or "Control group*" )

**S2: Categories of the factor hypothesis and the corresponding content**

| **Category** | **Content** |
| --- | --- |
| Human-animal interaction | The mentioning of “human-animal interaction” without further naming specific characteristic this interaction has or defining what elements of this interaction might lead to the suggested effects |
| Not specified | Not specified effects of different animal-assisted interventions, e.g., pet therapy, animal-assisted activities, horseback riding, animal-assisted interventions, animal-assisted reading program, animal-assisted psychotherapies etc. |
| Movement by the animal | Natural movement of a horse, rhythm/movement of horse |
| Social facilitator or catalyst | Animals as social catalyst, as social facilitator; animals can facilitate trust and bonding; companion animals facilitate social behavior; foster social communication and interaction skills; animal as catalysator for social learning |
| Relationship with an animal | Bond between human and animals; pet ownership; positive effects of human-animal bond; positive effects of relationship to animal; positive effects of attachment between human and animal |
| Other | Real life animal (1x); water (environment) (1x); biophilia (2x); non-verbal communication (1x); parental involvement (1x); influence of mount materials on the neuromuscular activation (1x); large animal (1x); insects can create nostalgic feelings (1x); concrete natural reinforcement for communication (1x); novel intervention (1x) |
| Presence of animal | Explicitly defining the presence of the animal to be responsible for the suggested effects |
| Physical contact | Physical contact (petting); tactile stimulation; activation of oxytocin system through interaction (physically); |
| Social or emotional support | Animal as an emotional social supporter; comparable to social support by a friendly person; social support from the presence of an animal |
| Taking care of an animal | Providing care to animals; animal enhances the role-taking abilities |
| Physical activity | Exercise with animals (e.g., walking a pet, exercise intervention with horses); horse riding |

**S3: Detailed information on the categories of the specific factors and the corresponding content**

| **Category** | **Content** | **Multiple categories applicable** |
| --- | --- | --- |
| Animal | Real-life animal, animal as factor; presence of animal |  |
| Interaction with animal | Playing with animal; response of animal; sound of animal; free interaction with dog (petting, playing etc.); individual chose of activity (grooming, petting, playing, teaching, walking on leash); exercise with animal (jumping over dog, crawling under dog etc.) |  |
| Movement by the animal | Riding; horseback riding; three-dimensional movement of horse |  |
| Physical contact | Petting |  |
| Taking care of an animal | Responsibility for animal; grooming an animal; training in animal care; feeding animal; brushing animal; working with farm animals; taking dog for a walk; rearing insects | Training in animal care |
| Training an animal | Giving animal commandos; teaching tricks and commandos; training in animal care | Training in animal care |
| Social interaction | Group activity; talking to another person (dog owner, researcher or trainer) |  |
| Other | Mount material (1x); Recreational/vacation atmosphere and therapeutic aspects (1x); distraction (1x); education about animal (2x); frequency (2x); familiarity (1x); therapeutic riding (1x); combination of activity and animal (1x) |  |
| Relationship with an animal | Bonding with animal; companionship; facilitate relationship between subject and animal |  |

**S4: Detailed information on the categories of the nonspecific factors and the corresponding content**

| **Category** | **Content** | **Multiple categories applicable** |
| --- | --- | --- |
| Therapeutic aspects | Occupational therapy; psychiatric treatment; physiotherapy; pharmacotherapy; kinesitherapy; therapeutic skiing; integrated psychological treatment; cognitive-behavioral intervention; psychotherapeutic treatment program; counseling; solution-focused therapy; aquatic-movement therapy; conventional play-based early intervention (PBI); attentional control intervention; empathy training; reality orientation therapy; social skills group psychotherapy; therapeutic skiing; pediatric examination; mindfulness-based intervention; medical treatment (e.g., dentist, venipuncture) | Physiotherapy; aquatic-movement therapy; exercises involving focus on the body and physical movement; psychoeducation; empathy training; academic-stress-management condition; kinesitherapy; therapeutic skiing |
| Social interaction | Group activity; talking to another person (e.g., dog owner, researcher or trainer) | Group sports; group training of social skills; being interviewed |
| Physical activity | Rehabilitation exercises; physical classes; physical activity; physiotherapy; home-based rehabilitation; group sports; movement of horse; riding; exercise group program; body and physical movements; stability exercises; treadmill; walking; therapeutic skiing; dance classes; aquatic-movement therapy; exercises involving focus on the body and physical movement; kinesitherapy | Group sports; physiotherapy; aquatic-movement therapy; physical-education classes; rhythm and music-based therapy; exercises involving focus on the body and physical movement; kinesitherapy; therapeutic skiing |
| Activity, distraction or absorption | Writing, reading alone; reading out loud to peers/ animal/ plush; recreation and occupation program; playing with toys/ peers; engaging in activities; watching movie; puzzle activity; recreational activities; playing; watching film of animal; focus view on living being (plant) or empty tank; exercises involving focus on the body and physical movement; educational and recreational activities; access to phone or tablet; drawing; being interviewed; distraction; cold pressor test | Exercises involving focus on the body and physical movement; educational and recreational activities; academic-stress-management condition; being interviewed |
| Education or training | Physical education classes; curriculum about pets and pet care; social skill training; horsemanship skills; coping skills education; lectures on healthy lifestyle choices; information about assistance dog; educational activities/ program; learning about caring for animals; content presentations and guided activities focused on enhancing self-regulation; learning about horses, attention exercise education; correctional & vocational programs; empathy training; educational and recreational activities; school lessons | Physical-education classes; psychoeducation; empathy training; group training of social skills; educational and recreational activities; academic-stress-management condition |
| Plush or toy animal | Plush animal; toy animal; robot | Condition with novel plush animal |
| Environment | Outdoor; water; aquatic movement; view on living being (plant) or empty tank; new setting/ environment; aquatic-movement therapy; farm, barn | Aquatic-movement therapy; focus gaze on living being (plant) or empty tank |
| Animal | Animal present; walking dog etc. |  |
| Interaction with something like an animal | Petting (plush animal); grooming and tacking stuffed toy horse; interaction with plush animal; grooming plush cat; horse riding simulator (HRS); simulated horseback riding; symmetrical sitting on stationary barrel | Horse-riding simulator (HRS); simulated horseback riding |
| Movement or rhythm | Rhythm and music-based therapy; movement on mechanic horse; vibration; auditory perception of beat based rhythms; audio consisted of the rhythmical beat-based sounds of horses | Rhythm and music-based therapy |
| Watching or /seeing animal | Exposure to pictures of the dog; visual of the animal; observation of dog-human interaction; watching film of animal | Watching film of an animal |
| Other | Bringing article to stimulate discussion (1x); sound of animal (1x); proximity effect (2x); talking about animals/pet (1x); horseback riding (not in a therapeutic context) (1x); taking care of something (1x); distraction (1x) |  |
| Relaxation | Sitting; relaxing; mindfulness-based intervention; taking a break during work | Mindfulness-based intervention |
| Novelty | Receiving a novel toy; condition with novel plush or toy to control for novelty effect | Plush animal; toy animal |
